# Predicting clinical response to anticancer drugs using an *ex vivo* platform that captures tumour heterogeneity

**DOI:** 10.1038/ncomms7169

**Published:** 2015-02-27

**Authors:** Biswanath Majumder, Ulaganathan Baraneedharan, Saravanan Thiyagarajan, Padhma Radhakrishnan, Harikrishna Narasimhan, Muthu Dhandapani, Nilesh Brijwani, Dency D. Pinto, Arun Prasath, Basavaraja U. Shanthappa, Allen Thayakumar, Rajagopalan Surendran, Govind K. Babu, Ashok M. Shenoy, Moni A. Kuriakose, Guillaume Bergthold, Peleg Horowitz, Massimo Loda, Rameen Beroukhim, Shivani Agarwal, Shiladitya Sengupta, Mallikarjun Sundaram, Pradip K. Majumder

**Affiliations:** 1 Mitra Biotech, Bangalore 560099, India; 2 Indian Institute of Science, Bangalore 560012, India; 3 Government Stanley Medical College, Chennai 600001, India; 4 Kidwai Memorial Institute of Oncology, Bangalore 560030, India; 5 Mazumdar-Shaw Cancer Center, Bangalore 560099, India; 6 The Broad Institute of The Massachusetts Institute of Technology and Harvard University, Cambridge, Massachusetts 02142, USA; 7 Brigham and Women’s Hospital, Harvard Medical School, Boston, Massachusetts 02115, USA; 8 Children’s Hospital, Boston, Massachusetts 02115, USA; 9 India Innovation Research Center, New Delhi 110092, India; 10 Harvard-MIT Division of Health Sciences and Technology, Cambridge, Massachusetts 02139, USA

## Abstract

Predicting clinical response to anticancer drugs remains a major challenge in cancer treatment. Emerging reports indicate that the tumour microenvironment and heterogeneity can limit the predictive power of current biomarker-guided strategies for chemotherapy. Here we report the engineering of personalized tumour ecosystems that contextually conserve the tumour heterogeneity, and phenocopy the tumour microenvironment using tumour explants maintained in defined tumour grade-matched matrix support and autologous patient serum. The functional response of tumour ecosystems, engineered from 109 patients, to anticancer drugs, together with the corresponding clinical outcomes, is used to train a machine learning algorithm; the learned model is then applied to predict the clinical response in an independent validation group of 55 patients, where we achieve 100% sensitivity in predictions while keeping specificity in a desired high range. The tumour ecosystem and algorithm, together termed the CANScript technology, can emerge as a powerful platform for enabling personalized medicine.

## Abstract (web summary)

Efficacy of anticancer treatments vary across patients, imposing a need for personalized approaches. Here the authors show that responsiveness to chemotherapy can be predicted using tumour explant cultures in a patient-matched microenvironment, coupled with a machine-learning algorithm.

## Graphical abstract

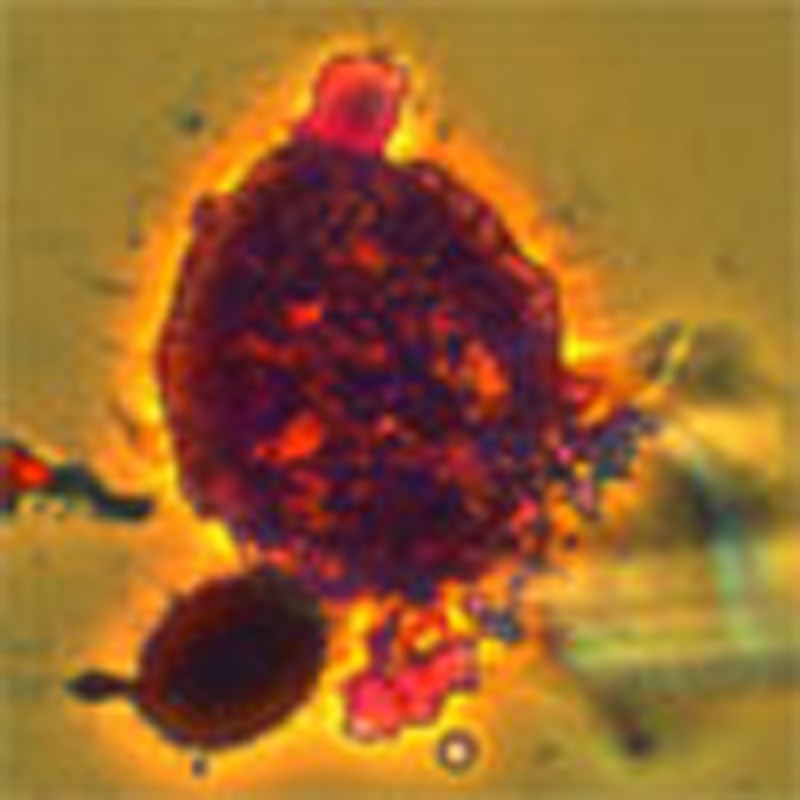

The ability to predict patient tumour response to cytotoxic or target defined therapeutic agents remains a holy grail. While molecular and genetic profiling is driving the evolution of subtype-specific personalized therapy^1^,^2^, the presence of a biomarker often does not translate into a successful clinical outcome^3^,^4^,^5^. For example, epidermal growth factor receptor (EGFR) inhibitors, cetuximab and panitumumab, are approved for metastatic colorectal carcinoma with wild-type *KRAS,* but provide clinical benefit in only 10–20% of selected patients^1^,^6^,^7^. A technology that can identify drug sensitivity and predict clinical benefit can significantly advance the clinical management of cancer.

Emerging evidence implicates intratumoral heterogeneity, both hierarchical and stochastic, in the variability of response to chemotherapy, which is not captured by the existing cancer cell biomarker-based approaches. Genetic and epigenetic distinctions within clonal populations could critically determine whether a particular drug combination will benefit a patient or result in resistance^8^,^9^,^10^,^11^,^12^,^13^. In addition, the contribution of the tumour microenvironment to these phenotypes is increasingly being appreciated^9^,^10^,^14^,^15^. Indeed, the spatial distribution of cancer and stromal cells within the tumour microenvironment can affect how they interact with each other and their microenvironment, which in turn can impact proliferation, differentiation, morphology and a range of cellular functions^16^,^17^,^18^. We rationalized that to predict the clinical outcome of chemotherapy with high accuracy, it is therefore important to conserve this clinical ‘global’ heterogeneity with high fidelity in terms of cancer and stromal cells, tumour microenvironment and architecture. Unfortunately, current gold-standard *in vitro* and *ex vivo* preclinical approaches that employ cell lines and spheroids^3^,^12^,^19^ or *ex vivo* organotypic tumour models are all limited by their inability to capture the full biological approximation of the native tumour, resulting in poor mapping to clinical outcomes^19^,^20^,^21^,^22^.

To create a clinically relevant predictive model, here we engineered an *ex vivo* tumour ecosystem, where thin tumour sections with conserved cellular and microenvironmental heterogeneity and architecture were cultured in tissue culture wells coated with grade-matched tumour matrix support in the presence of autologous serum (AS) containing endogenous ligands. The integration of the tumour ecosystems with a novel machine learning algorithm formed the CANScript platform, which reliably predicted the therapeutic efficacy of targeted and cytotoxic drugs in patients with head and neck squamous cell carcinoma (HNSCC) and colorectal cancer (CRC). The robustness of this platform in predicting clinical response could potentially be useful for personalizing cancer treatment.

## Results

### Role of matched tumour matrix proteins in CANScript platform

**Figure 1:**
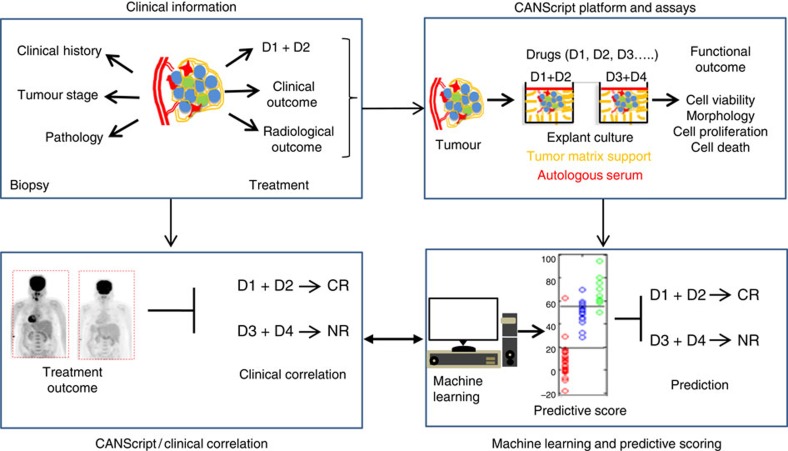
Schematic showing the development and validation of the CANScript technology. Four critical modules were integrated in generating and validating the CANScript platform. The first module involved collecting tumour core or surgical biopsy with tumour staging and pathology information besides clinical/treatment history. In the second module, tumour biopsy was rapidly processed into thin explants. Tumour biopsies were also used to generate either *in vivo* implants in mice, or processed for isolation and analysis of tumour matrix, which was used to develop the TMP cocktail. The explants were cultured in tumour- and grade-matched TMP and AS and incubated with selected drug regimens. While multiple drug regimens can be used, the one used by the oncologist for the patient was always included in the tumour explant culture. Functional outcome of treatment in terms of cell viability, pathological and morphological analysis, cell proliferation and cell death was quantified. In module three, these quantitative scores from the explants were aggregated using a machine learning algorithm to assign a final score, which helped rank the outcomes as CR,PR or NR. The learning algorithm was trained on data from 109 patients. In the final module, these predictions were tested against clinical outcomes from 55 new patients to validate the approach. D1, D2, D3 and D4 indicate different drug regimens.

**Figure 2:**
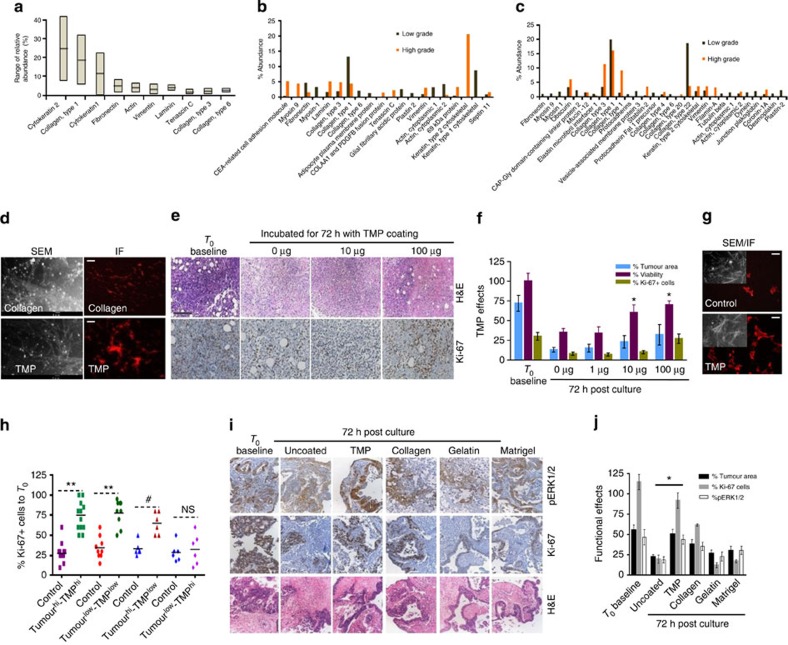
TMPs are critical for conserving primary tumour characteristics in explants. (**a**) The average composition and abundance range of key components of TMP. Abundance range was measured based on area under the peak using Pearson’s correlation for clustering of protein features. The line within each notch box represents the median, and the lower and upper boundaries of the box indicate first and third quartiles, respectively (*n*=24), of some of the key TMPs isolated from (**b**) HNSCC (*n*=12) and (**c**) CRC tumours (*n*=12) (**d**) scanning electron microscopy (SEM) images of plastic surface precoated with Collagen-I (top) or TMP cocktails (bottom). Scale bars, 1 μm. Adherence of the component proteins to the surface and their ability to form networks is shown following immunofluorescence (IF) staining using human Collagen-I antibody. Adherence was measured by detecting specific fluorescence signal in coated area contrasting to uncoated area of the same surface. Scale bars, 200 μm (right). (**e**) HNSCC explants were cultured for 72 h in plates coated with different concentrations of TMP as indicated. Maintenance of overall intratumoral heterogeneity and integrity was determined by hematoxylin and eosin staining (H&E; top) and tumour cell proliferation by Ki-67 staining (bottom). Scale bar, 100 μm. (**f**) Tumours from HNSCC patients were sliced. Explants were cultured for 72 h in plates coated with different concentrations of TMP as indicated. Percent tumour area, cell viability and Ki**-**67+ cells per field was measured (mean±s.d.). *P≤0.05 compared with uncoated control using paired *t*-test. Data represent one of the five independent experiments performed in triplicates. (**g**) HNSCC tumour slices cultured for 72 h with or without TMP were subjected to extraction of native extracellular matrix (ECM). Preservation of ECM 72 h post culture was determined by IF staining of extracted ECM parallel to SEM imaging (inset). (**h**) HNSCC tumours of high and low grades were sectioned cultured for 72 h in plates coated with matched and unmatched TMP (high and low grade) Scatter plot indicates the effects of grade-matched and unmatched TMP on retaining the proliferation profile. Percent Ki–67–positive cells from HNSCC explants were calculated at the end of 72 h based on *T*_0_ score. **P<0.0002, ^#^*P*<0.05 for the high-grade tumours cultured in presence of low-grade TMP by paired Student’s *t*-test. NS, not significant (*n*=12). (**i**) Representative images show the effects of CRC-specific TMP and other coating materials on pERK status (top), proliferation (middle) and morphology (bottom) of tumour explants. Scale bar, 100 μm. (**j**) Quantitative analysis of TMP on proliferation, tumour area and pERK status in CRC explants. **P <0.01 compared with T72 control (analysis of variance, *n*=8).

We depict the schematic for the development and validation of the CANScript platform in Fig. 1. A detailed patient demography and tumour subtypes used in this study are provided in Supplementary Table 1. As a first step towards mimicking the patient tumour ecosystem, we studied the contribution of cancer and grade-specific human tumour-stromal matrix proteins (TMPs) in preserving tumour morphology of HNSCC and CRC explants in an *ex vivo* setting. Indeed, three-dimensional (3D) matrix support is emerging as a critical factor that dynamically determines the fate of tumours in terms of integrity, survival, metastasis and response to chemotherapy^23^,^24^,^25^. We isolated and characterized the matrix components from clinical HNSCC and CRC tumours using processes described in detail in Supplementary Methods and Supplementary Fig. 1. The overall relative abundance of different TMP in tumour (both HNSCC and CRC) biopsies was analysed by liquid chromatography–mass spectrometry (LCMS/MS; Fig. 2a). Interestingly, a systematic analysis of the major TMP components not only revealed distinct compositions between the two tumour types and between high- and low-grade tumours of the same type (Fig. 2b,c), but also heterogeneity within the patient population as demonstrated using heat maps (Supplementary Figs 2a,d and 3a,d). Venn diagrams reveal unique matrix proteins that were conserved across the patient cohort within each tumour type and grade (Supplementary Figs 2b,e and 3b,e), which together with their abundance (median) (Supplementary Figs 2c,f and 3c,f) formed the basis for selection of the proteins to create the tumour- and grade-matched cocktails (listed in Supplementary Figs 2,3). We coated tissue culture microwells with these defined cancer- and grade-specific TMPs, which was confirmed using scanning electron microscopy and matrix proteins-specific immunofluorescence (Fig. 2d). Thin section tumour explants were then cultured in these TMP-coated wells. As compared with uncoated control, type- and grade-matched TMP showed a dose-dependent improvement in the maintenance of tissue morphology, proliferation and cell viability of the tumour explants (Fig. 2e,f). Furthermore, scanning electron microscopy analysis of native tumour extracellular matrix structure post culture indicated that integrity was better preserved in tumour explant tissues that were provided with TMP support (Fig. 2g). To further understand the role of grade-matched TMP cocktail, we did a cross-comparison analysis where high- and low-grade tumours were cultured in matched and unmatched TMP-coated plates. As shown in Fig. 2h, explants cultured on matched TMPs better retained native (T_0_) proliferation (Ki-67) state compared with the corresponding unmatched counterparts and no matrix controls. As expected, high-grade tumours did exhibit a greater capacity to preserve the proliferation profile even in low-grade TMP. Low-grade tumours in high-grade matrix performed poorly (Fig. 2h and Supplementary Fig. 4a). Next we compared the effects of different commercially available matrix proteins with TMP coating in maintaining the proliferation, viability and signalling activation of the explants to the native state (T_0_ baseline). As shown in Fig. 2i–j and Supplementary Fig. 4b, explants cultured in non-coated wells lost tumour architecture and exhibited decreased viability, proliferation and activation of oncogenic pathways compared with T_0_ baseline. While gelatin coating was no better than non-coated condition, collagen partially supported tumour proliferation, tumour area and phosphorylation of ERK1/2 but not cell viability. Interestingly, Matrigel, a widely used murine tumour-derived matrix, resulted in increased cell viability, tumour area and phospho-ERK but not in proliferation (Fig. 2j and Supplementary Fig. 4b). In contrast, explants cultured in matched TMPs retained tumour morphology, viability, proliferation and phospho-ERK1/2 status similar to the T_0_ baseline parameters. This observation is consistent with recent reports that highlight context-dependent stromal-epithelial interaction as a critical requirement of tumour cell survival and maintenance^10^.

### Autologous ligands maintain the signalling and phenotypes

**Figure 3:**
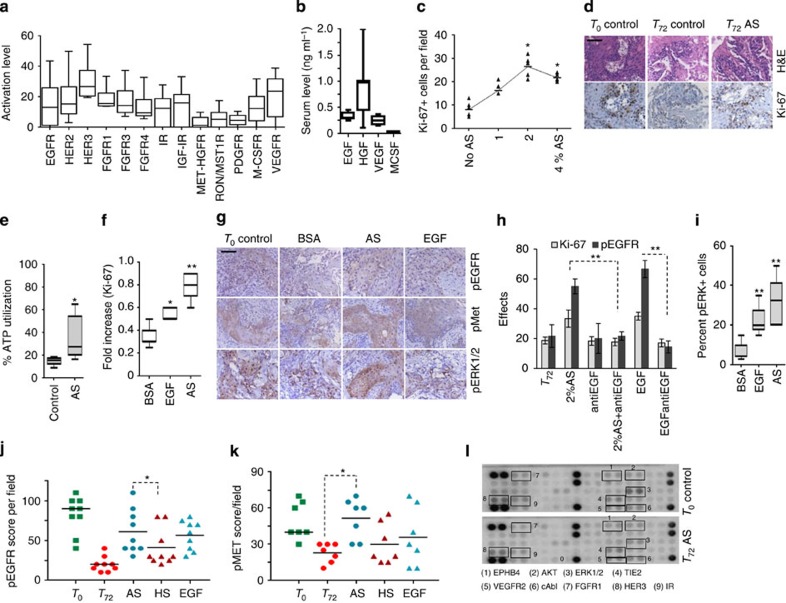
Autologous serum conserves the integrity of tumour explants. (**a**) Activation levels of major RTKs by RPPA profiling of patient tumours (*n*=5). Quantification of RTK activation was performed by measuring the signal intensity of individual analytes normalized to negative control. The line within each notch box represents the median, and the lower and upper boundaries of the box indicate first and third quartiles, respectively. Error bars (whiskers) represent the interquartile range. (**b**) Serum growth factor (EGF, HGF, VEGF and MCSF) profiles of HNSCC patients by ELISA (*n*=8). Horizontal line represents median and error bars indicate the interquartile range. (**c**) The dose-dependent effect of AS in HNSCC was measured by Ki**-**67. *P<0.001 by one way analysis of variance (ANOVA; *n*=9) compared with no AS control. (**d**) Tumour slices cultured in the presence or absence of autologous ligands for 72 h and stained with hematoxylin and eosin stain (top) and Ki**-**67 (bottom). Scale bar, 50 μm. (**e**) Box plot shows ATP utilization (*P<0.05 by *t*-test, *n*=6) at 72 h in the presence of AS. (**f**) Box plot shows fold increase in Ki**-**67-positive cells cultured with AS and EGF (*P<0.05 and **P<0.01, *t*-test, *n*=8). (**g**) Impact of AS on the balanced activation of different signalling receptors close to T_0_ baseline. Tumour explants were treated with 2% AS, 1 ng ml^−1^ per h EGF or 8% FBS+2% BSA (BSA Control) for 72 h. Tumours were stained for pEGFR (top), pMet (middle) and pERK1/2 (bottom). Scale bar, 100 μm. (**h**) Graph shows quantification of effects of different treatments on the proliferation and phosphorylated EGFR status in the explants. HNSCC samples were cultured in the presence of 2% AS or EGF up to 6 h for pEGFR and 72 h for detecting proliferation. Appropriate controls (no serum, no antibody and antibody alone) were included. Anti-EGF was added 1 h before stimulation. The effect was assessed by pEGFR and Ki**-**67 staining. All data (*n*=8) are represented as mean±s.d. **P<0.01 by *t*-test. (**i**) Box plot shows percent pERK positivity (*n*=8) **P<0.01 (by analysis of variance). Horizontal line represents median and error bars indicate the interquartile range. Graphs shows comparison of the capacity of AS, HS and EGF in activating (**j**) EGFR (*P<0.02 by *t*-test, *n*=9) and (**k**) in maintaining phospho-Met expression (*P<0.0001 by *t*-test, *n*=7). (**l**) Global RTK profiles of cultured HNSCC tumour explants and corresponding T_0_ baseline was compared following stimulation with 2% AS for 72 h. Total cell lysates were applied to array slides precoated with different antibodies against RTK pathways. Signal was detected by chemiluminiscence method.

A heterogenous tumour microenvironment represents a diverse network of oncogenic signalling pathways, which are activated in both ligand-dependent and -independent manner and can spatiotemporally and dynamically cross-talk^26^,^27^,^28^,^29^,^30^. Indeed, a reverse phase phosphoprotein array (RPPA)-based profiling of key receptor tyrosine kinases (RTKs) and their nodal proteins in the tumour biopsies revealed a heterogeneity in the baseline activation levels of these receptors and downstream signals (Fig. 3a and Supplementary Fig. 5a,b). This led us to hypothesize that a balanced induction of these receptors using their original ligands in an individualized setting is critical to mimic the baseline networks of the parent tumour *ex vivo*.

Autocrine–paracrine loops of growth factors enriched in patient sera contribute to the activation of signalling networks and survival cascades in cancer cells^10^,^31^,^32^. As the second step towards fabricating the CANScript platform, we therefore studied the functional attributes of AS. As shown in Fig. 3b a number of growth factors (represented by EGF, hepatocyte growth factor (HGF), vascular endothelial growth factor (VEGF) and macrophage colony-stimulating factor (MCSF)) were found to be within clinically detectable ranges in patient sera. The variability in the levels of these growth factors that exists between individuals further underlined the importance of using the complete AS for a balanced induction of signal transduction pathways as opposed to an artificial combination of growth factors. We first performed a dose–response analysis, where increasing concentrations of AS was used in combination with a reducing percentage of fetal bovine serum (FBS) in the culture for 72 h. A concentration dependent increase in cell proliferation in the explants was observed while supplementing the system with AS that attained the peak at 2% (Fig. 3c). Concomitantly, 2% AS also mimicked the native state (morphology and proliferation) of tumours at T_0_ baseline (Fig. 3d). The decline above this concentration is consistent with earlier observations with growth factor ligands and possibly arises due to the downregulation of targets^33^. In addition, 2% AS (+8% FBS) resulted in significant increase in ATP utilization and cell proliferation compared with 10% FBS or recombinant EGF alone (Fig. 3e,f). Furthermore, compared with exogenous EGF controls, the addition of AS significantly preserved the major signalling networks as measured by phosphorylation of EGFR, Met and downstream target, ERK1/2. It is interesting to note that 1 ng ml^−1^ concentration of EGF predominantly activates EGFR pathway alone. In contrast, 2% AS showed the capacity to activate both EGFR and HGFR/Met pathways along with downstream ERK1/2 comparable to the T_0_ baseline, consistent with the balanced effect of patient-derived ligands in its natural milieu. The enhanced response to AS was reduced to T_72 h_ baseline (that is, no AS control) using neutralizing antibodies to EGFR, which is consistent with the aberrant activation of EGFR pathway in a majority of HNSCC and other cancers of epithelial origin^27^,^29^. However, the neutralizing antibody failed to fully abrogate the proliferation below the level of T_72 h_ control, suggesting that despite the predominant role of EGFR in some individual tumours additional constitutive mechanisms exist that might contribute to minimal maintenance of these tumours (Fig. 3g–i). It is obvious that the survival of tumour is not a consequence of dependency on single pathway lineage or network.

To further validate the contribution of autologous sera in personalizing the explant culture, we compared the individual effects of heterologous/allogenic sera (HS) obtained from treatment naïve patients (age, sex and cancer-type matched) with AS and recombinant EGF. As shown in Fig. 3j,k, while EGF resulted in the maximum effect in inducing EGFR phosphorylation, 2% AS efficiently maintained both EGFR and Met phosphorylation. In contrast, 2% HS, while exerting a greater effect than no ligand (T_72 h_) control, was significantly inferior to AS. Similar pattern was observed for Ki-67 (Supplementary Fig. 6a). Taken together, these results indicate that presenting the entire repertoire of growth-promoting ligands by using AS is critical to fully capture the parental activation status of important receptors in the personalized explant setting. Indeed, RPPA array-based analysis of the parent HNSCC tumours (T_0_ baseline) showed that a bulk of the proteins in RTK cascades that were upregulated are largely conserved in the tumour explants cultured in 2% AS (Fig. 3l and Supplementary Fig. 6b).

### Reconstructing a tumour ecosystem

**Figure 4:**
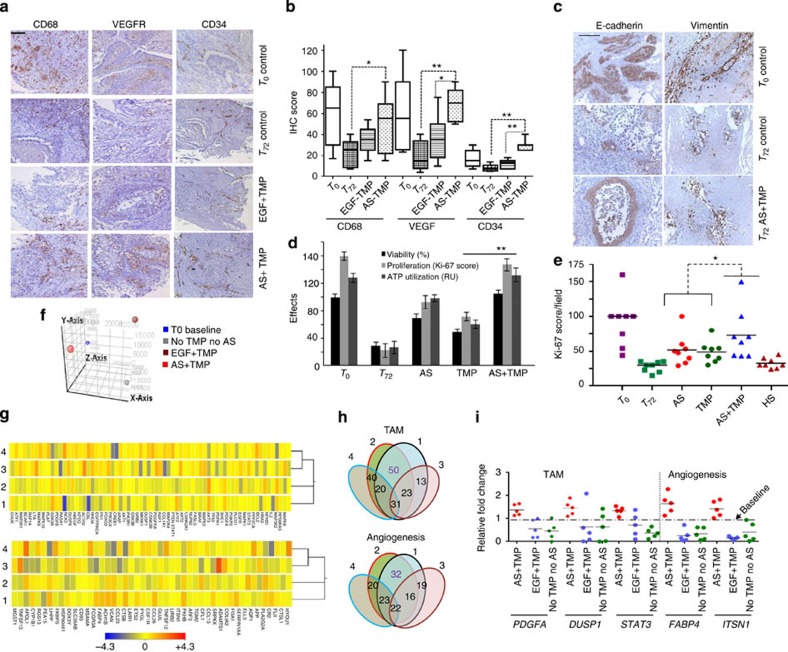
Integration of both TMP and AS in the CANScripts maintains the tumour ecosystem. (**a**) Representative IHC images show the effect of AS and matchedTMP on the phenotypic stability of tumour explants 72 h post culture. Tumour sections were stained for CD68, VEGFR and CD34. Scale bar, 100 μm. (**b**) Quantitative IHC based box plot indicates CD68, VEGFR and CD34-positive cells in the explants maintained under different conditions. Horizontal line represents median and error bars indicate the interquartile range. *P<0.05 and **P<0.001, respectively (paired *t*-test, *n*=8). (**c**) Representative IHC images show EMT related markers of tumour microenvironment in the CANScript explants. Scale bar, 100 μm. (**d**) Graph shows the combined effects of AS and TMP on the functional integrity of the explants. Tumour sections were cultured for 72 h. Number of Ki**-**67-positive cells were counted and plotted along with percent viability and ATP utilization per section in triplicates (mean±s.d.). **P< 0.01 (by analysis of variance). (**e**) The combined effects of AS and TMP on the functional integrity of explants are represented as scatter plot (*n*=8). Number of Ki**-**67-positive cells were counted and plotted. HS was run as a control. *P< 0.05 (by paired *t*-test). (**f**) 3D-PCA plot showing global gene expression patterns between different culture conditions (that is, no AS and no TMP, EGF+TMP, AS+TMP and T_0_ baseline) obtained from HNSCC tumour explants after 12 h. After initial normalization of data analysis was performed compared with baseline. (**g**) Heat map analysis of the microarray data showing the genes related to TAM (top) and angiogenesis (bottom). Tumours explants were cultured in TMP-coated plates with AS (AS+TMP, lane 2) or EGF (EGF+TMP, lane 3) or in uncoated plates without AS (No TMP and no AS Control, lane 4) and transcriptomic pattern was compared with base line tumour (lane 1). Heat map scale indicates the expression range. Clustering of genes was performed by k- means algorithm. Distance was measured by Euclidean distance metric. (**h**) Venn diagram showing number of overlapped genes related to TAM and angiogenesis between the three culture conditions. (**i**) Validation of microarray gene signature by qRT–PCR for TAM (left) and angiogenesis (right); selected genes from each signature was run in triplicates (technical replicates) normalized to baseline expression (biological replicates) and compared between conditions as indicated in the scatter plot (*n*=5).

As the final step towards constructing the CANScript tumour ecosystem, both conditions (that is, TMP and AS) were contextually integrated in the explant system. Immunohistochemistry (IHC) labelling was used to evaluate a number of static and dynamic phenotypic markers associated with functional heterogeneity of tumour microenvironment. Profiling for CD68 (marker for immune component)^34^, VEGFR (marker for angiogenesis), CD34 (marker for angiogenesis and progenitors)^35^, E-Cadherin and Vimentin (markers for epithelial mesenchymal transition (EMT)) revealed that the combination of AS and TMP conserved the parental (T_0_) phenotypes better than T_72 h_ control or EGF+TMP(Fig. 4a,b). Similar effects of AS and TMP were also observed for EMT-specific markers (Fig. 4c). Furthermore, cell viability, proliferation index, and metabolic state of the explants in the CANScript tumour ecosystem was similar to native (parent/T_0_ baseline) tumour and significantly enhanced compared with control explants cultured without AS and TMP, or with either AS or TMP alone. The pattern of augmentation of Ki-67 upon AS+TMP was found to be consistent and significant (Fig. 4d,e). Together these results indicate that the native tumour-stromal micro-architecture and phenotypic features were largely conserved in the CANScript tumour ecosystem compared with the culture conditions with only TMP or AS or EGF-supplemented TMP. Next, we used microarray profiling to compare the transcriptome of primary tumours at baseline (T_0_) and serially sectioned tumour explants cultured under different conditions. Indeed, a high degree of conserved global transcriptomic profile consistent with the primary tumour was observed only in the case of the CANScript platform that integrated both TMP and AS, while supplementing the explant cultures with either AS or TMP(+EGF) alone resulted in distinct transcriptomic signatures (Fig. 4f,g). Concurrent to the phenotypic expression as shown in Fig. 4a,b, conservation of stromal gene expression signatures, specifically linked to tumour-associated macrophages and angiogenesis were also observed (Fig. 4g). To further confirm these results, a selected panel of genes relevant to TAM (that is, *PDGFA*, *DUSP1* and *STAT3*) and angiogenesis (that is, *FABP4* and *ITSN1*) signatures (Supplementary Table 2) was analysed under different conditions using qRT–PCR. As shown in Fig. 4i, the expression of these markers were conserved only under AS+TMP condition but not when either is absent. In addition, expression of tumour-associated key cytokine/chemokines, such as interleukin-6, interleukin- 8 and CXCR-4, matrix degrading enzyme matrix metallopeptidase 9 (MMP-9) and cancer stem cell markers like CD44 and ALDH1 observed in the parent HNSCC tumours were also preserved in the CANScript tumour ecosystem (Supplementary Fig. 7a–c). It is important to note that unlike common synthetic organotypic inserts, the CANScript platform exhibited enhanced preservation of native tumour morphology and proliferation status (Supplementary Fig. 7d). Taken together, these results suggest that a number of phenotypic markers characteristic of EMT, immune cells and cytokines as well as cancer stem cell phenotypes are more consistently and collectively better conserved in this platform compared with culture conditions with either TMP or AS or EGF-supplemented TMP.

### CANScript predicts response to cytotoxic and targeted drugs

**Figure 5:**
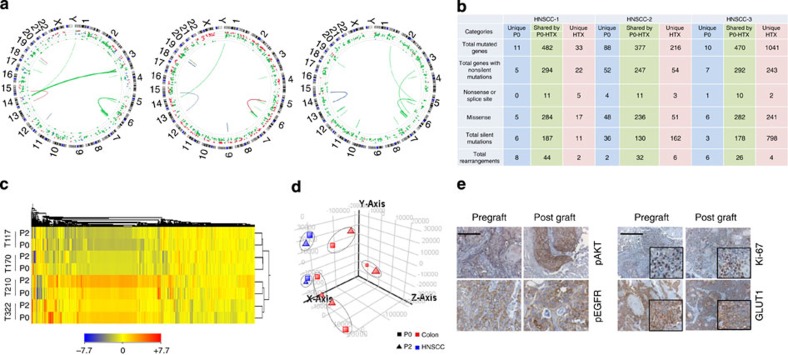
Comparative profiling of patient tumours and their corresponding xenografts. (**a**) Mutational and translocation spectrum obtained from the whole-exome sequence analysis (Agilent 44 Mb, × 50 coverage) of HNSCC patient tumours and also their corresponding xenograft tumour (passage no.2) tissues. In representative Circos plots each dot represents a mutation and line represents translocation. Blue colour stands for original tumours, green colour stands for overlapped original and xenografted tumours and red colour denotes the events in xenograft only. (**b**) Exome data table illustrates that the HNSCC tumours when passaged in mice (P2) retain majority of genomic characteristics of the baseline tumour. (**c**) Unsupervised 2D hierarchical clustering performed on colon samples shows that expressed genes in primary tumour (P0) are associated with HTX and stably expressed when passaged in mice (P2). Scale represents expression ranges (fold normalized changes, bottom). (**d**) 3D**-**PCA plots generated by GeneSpring GX software to show the clustering of samples of same origin and serial passage. The plot shows six distinct clusters comprising of four pairs of colon carcinoma and two pairs of HNSCC samples. (**e**) Representative IHC images of early passages of HTXs and matched primary tumours. Primary HNSCC tissues (pregrafts) were propagated up to passage 2 (post grafts) in SCID mice. Tumours from both pregrafts and post grafts were stained with anti–Ki–67 (right, top), antibodies against pAKT (left, top) and pEGFR (left, bottom). Scale bars indicate 100 μm for Ki-67 and pAKT and 50 μm for pEGFR. Tumours from both pre- and post grafts were also stained for expression of GLUT1 (right, bottom) using specific antibodies. Scale bars, 100 and 50 μm (inset).

**Figure 6:**
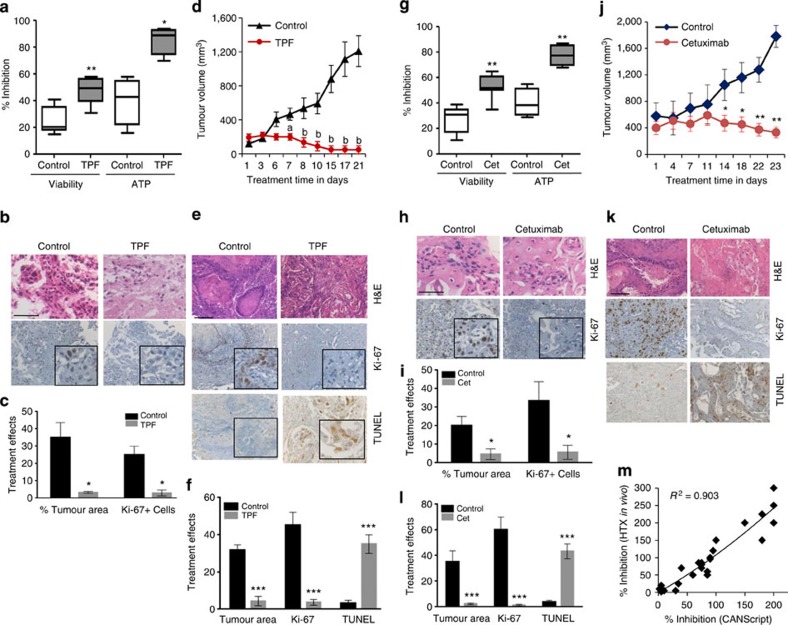
Application of CANScripts in functional *ex vivo* and *in vivo* correlation. (**a**) Antitumour effects of TPF chemotherapy regimen on HNSCC tumour explant culture. Box plots show inhibition of viability and ATP utilization in TPF-treated tumours as calculated using T_0_ values as baseline. Horizontal line represents median and error bars indicate the interquartile ranges. *P<0.01 and **P<0.001 compared with vehicle-treated control (*n*=20) by *t*-test. (**b**) Representative images show corresponding IHC profile. Tumour sections were stained with H&E (hematoxylin and eosin stain; top) and Ki-67 (bottom). Scale bar, 50 μm. (**c**) Graph shows percent tumour area and Ki-67-positive cells from vehicle- and TPF- treated explants. Data shown are mean ±s.d. *P<0.05 by *t*-test (*n*=3). (**d**) Graph shows *in vivo* tumour growth inhibition in xenografts following TPF treatment till 21 days of treatment. Data shown are mean tumour volume ±s.d. with six mice per group (^a^*P*<0.01 and ^b^*P*<0.001 by analysis of variance, ANOVA) compared with vehicle control. (**e**) Representative IHC images of pre- and post-treatment tumours stained with H&E stain (top), Ki-67 antibodies (middle) and TdT-mediated dUTP nick end labeling (TUNEL; bottom). Scale bar, 100 μm. (**f**) Graph shows quantitative analysis of IHC for tumour area, Ki-67+ cells and TUNEL+ cells from control and TPF treatment. Data are mean±s.d. of six mice per group. ***P<0.001 compared with vehicle control (paired t-test). (**g**) Cetuximab efficacy in HSNCC CANScripts. Explants were treated with DMSO or cetuximab. Box plots represent percent inhibition of cell viability and ATP utilization (*n*=20). Horizontal line represents median and error bars indicate the interquartile range. **P<0.001 by *t*-test. (**h**) Representative images of tumour sections labelled with H&E for morphology (top panel) and anti-Ki-67 antibodies for proliferation (bottom panel). Scale bar, 50 μm. (**i**) Graph shows quantification of effects of treatment in the CANScripts in terms of percent tumour area and Ki**-**67-positive cells from control and cetuximab treatment. Data represented as mean ±s.d. (*n*=3). *P<0.001 by *t*-test. (**j**) Graph shows tumour growth inhibition in cetuximab-treated mice. Data are mean tumour volume±s.d. *n*=10. *P<0.02 and **P<0.001 (ANOVA) versus vehicle control. (**k**) Representative IHC reveals changes in H&E (top), Ki**-**67 (middle) and TUNEL (bottom). Scale bar, 100 μm. (**l**) Quantitative analysis of tumour area, Ki**-**67 and TUNEL from control and cetuximab-treated mice. All data indicate mean ±s.d. ***P<0.001 (*t***-**test). *n*=6. (**m**) The correlation observed between the efficacy data from TE explants and *in vivo* studies. *R*^2^ was calculated using Spearman’s correlation coefficient method.

The conservation of patient tumour heterogeneity in the CANScript tumour ecosystem prompted us to explore the possibilities of using this as a preclinical tool to predict anticancer drug response. To assess this, we first compared drug response in human tumour-derived xenotransplants (HTX) and in matched CANScripts explants (constructed from passage 2, that is, P2-HTX). Primary HNSCC tissues were propagated in severe combined immunodeficiency mice up to second passage (P2-HTX). Since response and resistance to a particular drug combination can be intrinsically controlled by deregulation at the genetic and epigenetic levels,^11^,^36^,^37^,^38^ we first mapped the degree to which a xenotransplanted tumour (at P2) conserves the descriptors of the primary tumour. Interestingly, exome data from three different primary samples, HNSCC-1, HNSCC-2 and HNSCC-3, and their matched P2-HTX, showed that while the overall events of mutation and translocation of primary tumours were largely preserved when passaged in immunocompromised mice, there were mutations that were unique to original parental P0 and P2-HTX, respectively (Fig. 5a,b and Supplementary Table 3). However, global transcriptome pattern showed a good association between P0 and matched HTXs (Fig. 5c,d). Furthermore, histopathological characterization of P2-HTX revealed that the HTX successfully conserved key morphological and molecular characteristics of original parental (P0) tumours, including the expression of proliferation marker (Ki-67), glucose transport (GLUT1), phospho-EGFR and phospho-AKT (Fig. 5e). Subsequently, these extensively characterized P2-HTX were used as surrogates for initial functional validation of the CANScripts. HTX-derived CANScripts were concurrently treated with the clinically approved cytotoxic drug regimen of docetaxel, cisplatin and 5-fluorouracil (TPF), segregated into two groups of responders and non-responders based on viability, ATP utilization, proliferation status and loss of tumour area/nuclear fragmentation (Fig. 6a–c and Supplementary Fig. 8a–c). Interestingly, we noticed an excellent correlation between the outcomes in the CANScript platform and the response to chemotherapy in the HTX studies. For example, cases predicted as responders using the CANScript tumour ecosystem mapped to a significant inhibition of tumour growth when the animals were treated at maximum tolerated dose daily for up to 21 days (Fig. 6d). The results were further validated at the molecular level by determining the end point changes in mean tumour area/nuclear size in sections, Ki-67 and concomitant drug-induced increase in apoptotic cells by staining with TUNEL method (Fig. 6e,f). Similarly, cases predicted as non-responders using the CANScript tumour ecosystem did not show any effect in HTX system, as defined by the lack of any distinctions in Ki-67 and active Caspase-3 expression between the treated and untreated groups (Supplementary Fig. 8a–e).

The *ex vivo* to *in vivo* correlation in response to a general cytotoxic drug combination that we observed in HNSCC samples encouraged us to further validate the predictive ability of CANScript for targeted therapeutics. For this purpose we used HTXs generated from HNSCCs harbouring wild-type or mutant *KRAS*. Consistent with the results observed earlier with cytotoxics, a positive response in the CANScript explants with cetuximab (Fig. 6g–i) was mirrored by tumour inhibition *in vivo* (Fig. 6j–l).The functional outcome was correlated with a decrease in Ki-67 positivity, increased TUNEL and a reduction in phospho-EGFR levels in both the CANScript explants and *in vivo* (Fig. 6h,i,k,l and Supplementary Fig. 9a,b). We next tested the effect of cetuximab in HTX and CANScript explants generated from CRCs. As shown in Supplementary Fig. 10a–g, an inhibitory outcome in the CANScript explants correlated with a significant tumour growth inhibition *in vivo*, while in the absence of an inhibitory effect in the tumour ecosystem (TE), minimal tumour growth inhibition was evident *in vivo* (Supplementary Fig. 10h–n). In the cetuximab-treated groups, responders showed a decrease in Ki-67 and phospho-ERK levels and increase in cleaved caspase-3 expression (Supplementary Fig. 10b,c,e–g). This was not evident in the non-responders (Supplementary Fig. 10i,j,l–n). Collectively, we observed a linear correlation (*R*^2^=0.903, *n*=26, by Spearman’s correlation coefficient) between CANScript explants outcomes and *in vivo* HTX responses (Fig. 6m).

### CANScript as a tool to predict treatment outcome in patients

**Figure 7:**
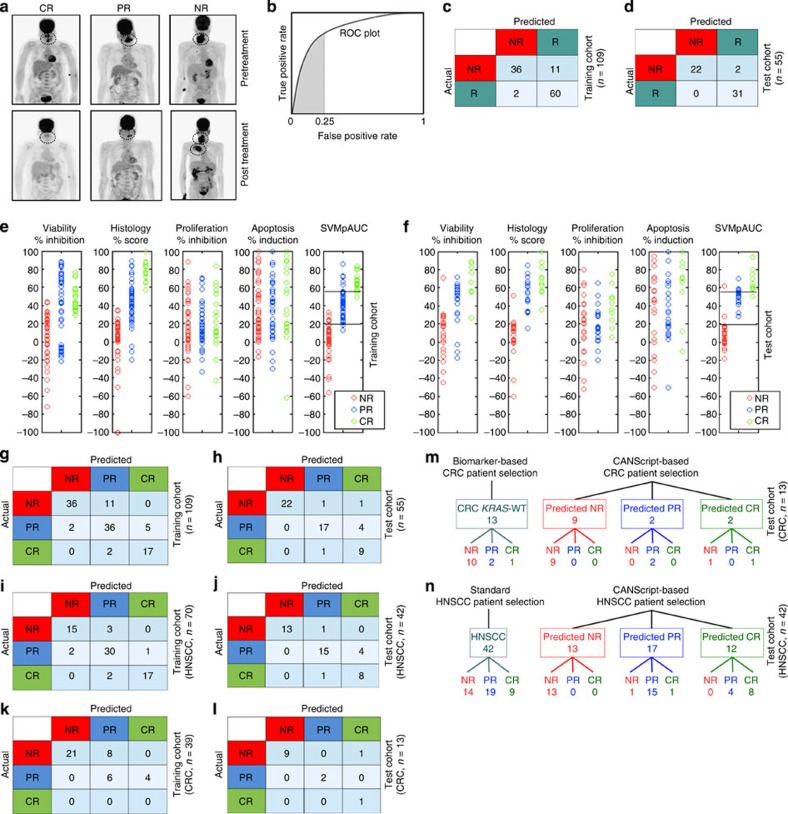
Validation of CANScript platform using clinical data. (**a**) Positron emission tomography–computed tomography (PET–CT) for representative cases of CR (left), PR (middle) and NR (right) as determined by PERCIST. Primary treatment-naïve HNSCC patients underwent FDG–PET–CT scan examination before (predose) and after three cycles of TPF treatment (post dose). Clinical response to the drugs for individual patients was evaluated based on PERCIST data. (**b**) ROC plot showing true positive rate (sensitivity) and false positive rate (one minus specificity); the shaded area represents the partial area under the ROC curve up to false positive rate 0.25. The SVMpAUC algorithm used to learn a NR/R model to distinguish the non-responders from responders maximized the partial area under the ROC curve up to false positive rate of 0.25 on the training set. This encourages learning a model with high sensitivity, minimizing the number of potential responders (PR or CR patients) that are predicted to be NR while keeping specificity at least 75%. (**c**) Performance of learned NR/R model on the training set. Confusion matrix displays the number of patients with various actual and predicted responses to TPF for HNSCC and cetuximab+FOLFIRI for CRC in the training set (*n*=109). (**d**) Performance of learned NR/R model on the test set. Confusion matrix displays the number of patients with various actual and predicted responses in the test set (*n*=55). (**e**) Plots showing values of the functional read-outs from the CANScripts (that is, viability, histology, proliferation and apoptosis), as well as scores assigned by the SVMpAUC-learned model to patients in the training set, and (**f**) in the test set. (**g**) Performance of final refined NR/PR/CR prediction model on the training set. Confusion matrix displays the number of patients with various actual and predicted responses to TPF for HNSCC and cetuximab+FOLFIRI for CRC in the training set. (**h**) Performance of final refined NR/PR/CR prediction model on the test set. Confusion matrix displays the number of patients with various actual and predicted responses in the test set. (**i**,**j**,**k** and **l**) Performance of final refined NR/PR/CR prediction model on HNSCC cases alone in the training and test sets, and on CRC cases alone in the training and test sets, respectively.(**m**) CANScript-based model is a better tool than biomarker- *(KRAS)* based prediction of response to cetuximab and FOLFIRI in CRC. (**n**) CANScript-based model is a better tool than standard patient selection for response to TPF in HNSCC.

The concordance in outcome between HTX *in vivo* and corresponding CANScript studies suggested the possibility of using the latter for predicting the treatment outcome in patients. The CANScript explants were generated from biopsies of CRC and HNSCC tumours from 109 patients and were incubated with the same drug combination as that administered to the patient, that is, docetaxel, cisplatin and 5-fluoro uracil (5-Fu) for the 70 HNSCC patients and cetuximab+FOLFIRI for the 39 CRC patients. The functional read-outs from these CANScripts, quantified in terms of viability, histopathology, proliferation and apoptosis, together with the observed clinical response in the matched patients, classified as progressive disease/non-response (NR), partial response (PR) or complete response (CR) based on PERCIST guidelines (Fig. 7a), were then used as the training set for a novel machine learning algorithm. In this algorithm, as the first step, we classified patients as simply responders or non-responders, with a focus on ensuring high sensitivity (true positive rate). This was formulated by maximizing the partial area under the receiver operating characteristic (ROC) curve (partial area under the curve (AUC)) up to an acceptable false positive range (Fig. 7b). To this end, PR and CR were grouped together into a responder (R) category and a linear prediction model was learned using SVMpAUC, a recently proposed structural support vector machine algorithm for optimizing partial AUC. The learned model was designed to maximize partial AUC while achieving at least 75% specificity (that is, at most 25% false positive rate) on the training set, and assigned coefficients of 0.2977, 0.5562, 0.0073 and 0.1388 to the viability, histology, proliferation and apoptosis read-outs, respectively, together with a threshold of 19.1 (that is, cases assigned a weighted score >19.1 by the learned model were predicted to be responders).The model achieved 96.77% sensitivity on the training set (Fig. 7c). We then tested the learned algorithm on a new test group of 55 patients, consisting of 42 HNSCC and 13 CRC patients treated with the same drugs as above, where the model achieved 91.67% specificity and 100% sensitivity (Fig. 7d). In particular, no potential responders (PR or CR patients) in the test set were predicted as NR (Fig. 7d).

In the next step, the learned model was refined to classify the predicted responders into partial and complete responders (PR and CR), by selecting a threshold that maximized PR versus CR prediction accuracy on the training set. Following this, scores between 19.1 and 55.14 were classified as PR, and those >55.14 as CR. As can be seen in Fig. 7e,f, the coefficients assigned to the four read-outs by the SVMpAUC-learned model, together with the above thresholds, resulted in predictions that were significantly better than what could be achieved by predicting using any one of the functional read-outs alone. Confusion matrices summarizing predictions in each category on both the training and test sets are shown in Fig. 7g,h; break-ups among HNSCC and CRC cases are shown in Fig. 7i–l. The resulting predictions had 87.27% accuracy on the test set (Fig. 7h). In particular, among the 55 test cases, there were only seven prediction errors: four PRs were predicted as CR; one CR was predicted as PR; one NR was predicted as PR; and one NR was predicted as CR (Fig. 7h). This is the benefit of using the SVMpAUC machine learning algorithm, which explicitly encourages high sensitivity in the learned model (indeed, a standard support vector ordinal regression algorithm which directly classified the patients into one of the three categories yielded a lower accuracy of 81.82% on the test set, making a total of 10 prediction errors on the 55 cases, which included 1 PR case predicted as NR). Again, it is worth emphasizing that these errors using the SVMpAUC machine learning algorithm were all ‘benign’, in that no potential responder (PR or CR) was predicted as a NR. While such ‘benign’ errors do mean unwarranted drug use that can result in potential side effects, it also means that no patient who would respond to chemotherapy is denied a drug based on a false prediction. Indeed, current clinical practice also assumes this principle, where the error rate is significantly higher as seen in our study. For example, as shown in Fig. 7m, biomarker analysis selected all 13 CRC patients in the test set, all of whom were positive for wild-type *KRAS,* to receive cetuximab. However, as can be seen, only 3 of these 13 wild-type *KRAS* patients actually responded to the drug (1 exhibited CR and 2 exhibited PR), while the remaining 10 presented with progressive disease. Interestingly, the CANScript platform predicted two CRs, two PRs and nine NRs, with only one actual NR case being wrongly predicted as CR. As shown in Fig. 7n, based on standard practice, all 42 HNSCC patients in the test set received TPF. However, 14 of these patients did not respond to the drug combination. The CANScript platform could identify 13 of these as NRs. Again, importantly, all patients predicted by the platform as NRs were indeed NRs. It should be noted that 13 and 42 are small sample sizes, and that larger-scale studies are needed in the future to establish similar results on larger sample sizes; however based on the observed improvements over the standard/biomarker-based approach, we anticipate that the CANScript platform can emerge as a powerful strategy for predicting chemotherapy outcomes.

## Discussion

While biomarker driven personalized cancer therapy has emerged as a powerful concept, the mere presence of a biomarker in a cancer cell may not translate into clinical efficacy^1^,^6^,^7^,^39^. This arises from heterogeneity, where multiple genetic, epigenetic and phenotypic alterations along with immune and metabolic changes represent a complex state of the neoplastic transformation^40^. Indeed, in the current study, of the 52 patients who received cetuximab based on wild-type *KRAS* status, only 1 exhibited CR and 12 exhibited PR, and the remaining 39 presented with progressive disease. While the use of more than one biomarkers, for example, the use of wild-type *KRAS* and *BRAF* to select patients eligible for cetuximab^41^ is the emerging trend, the ability to predict chemotherapy outcomes accurately at an early time point still remains a holy grail in the management of cancer. Here we have demonstrated the development of a novel technology platform that integrates a comprehensive explant culture with a machine learning algorithm to better predict chemotherapy outcomes. As we have demonstrated in this study, the CANScript platform is versatile in its ability to predict the outcomes of both cytotoxic chemotherapy regimens and targeted therapeutics.

A key attribute of the CANScript platform is its ability to capture the intratumoral heterogeneity to a greater degree than achieved by biomarker-based selection of cancer cells. Cancer stem cells, stromal cells such as intra and peritumoral immune cells, and vascular components can further add to the heterogeneity and contribute towards tumour survival, progression and metastasis^15^,^17^,^34^,^42^, suggesting that an explant culture that globally conserves these distinct cellular components in their original architecture, as evident in the CANScript platform, is important for increasing the probability of predicting a chemotherapy outcome. Indeed, anticancer drugs have been reported to exert their effects by altering both cancer cells and tumour microenvironment^10^,^11^,^34^,^40^.

Interestingly, short-term cultivation of primary explants of human tumours had been explored previously, for example, growing the specimens in plasma clots^43^ or using engineered 3D explant cultures^20^,^25^,^44^,^45^,^46^,^47^,^48^. While these explant models did capture the heterogenous cancer and stromal cell population to certain degree, and were used to study tumour heterogeneity, invasiveness and response to treatment^15^,^16^,^17^,^21^,^25^,^49^, these attempts did not show full viability at diverse functional levels. While extracellular matrix support was shown to help preserve and recreate many important morphologic and phenotypic properties in these 3D spheroids and organotypic cultures, these studies did not elucidate the importance of conserving tumour type- and grade-matched matrix factors in maintaining functional organization and dynamics. Indeed, our results indicate that the composition of TMPs is distinct between tumour types and also between grades. Nor did these studies recreate the oncogenic signalling networks encompassing the activation of diverse RTK signalling with extensive heterogeneity and cross-talks^20^,^27^,^30^,^50^,^51^,^52^. Importantly, our results with mismatched matrix or HS controls indicate the criticality of a matched tumour microenvironment together with AS in preserving the phenotypic and molecular features of the native tumour. It is evident from the RPPA profiling of key signalling pathways and physiologically relevant growth factors detectable in patient serum that extensive heterogeneity exists between patients and that a truly personalized milieu with an active balance of multiple parallel signalling cascades can therefore be successfully created by AS^53^,^54^,^55^,^56^,^57^. It should, however, be noted that while AS and TMP independently and collectively improved explant culture quality, not all aspects are necessarily dependent on dual presence of AS and TMP. For example, growth factor dependent features are better sustained in presence of AS, whereas TMP plays a dominant role in tumour heterogeneity and at the phenotypic level in maintaining survival and proliferation.

The ability to predict outcomes is not only attractive from a clinical perspective, but also has major implications on preclinical cancer research, where the focus has been to develop assays that can bridge the translational gap. While animal models have been used as the front line in predicting efficacy, the predictive value of these models is debatable, a consequence of using cell lines cultured over years that are no longer representative of the original tumour. Furthermore, transgenic murine models may recapitulate a specific cancer pathway, but fail to capture the true heterogeneity that is characteristic of human tumours. For example, we observed in our study that while EGFR generally plays a critical role in HNSCC, additional driver mechanisms such EphB4, AKT, ERK1/2, Tie2, VEGFR2, cAbl, FGFR1, HER3 and IR are activated. Indeed, such stochastic heterogeneity has been implicated in the induction of adaptive resistance. There is therefore a resurgence in the use of early-passage patient-derived xenografts for predicting clinical responses. Consistent with these recent studies, we observed a good concordance in terms of histopathology and gene expression between the tumour biopsy (P0) and the 2ndpassage xenografts (P2-HTX). However, we did observe unique mutations between the P0 biopsy and the P2-HTX xenografts. It is possible that these differences between P0 and P2-HTX arise due to intratumour heterogeneity at the time of implantation^50^. In our study, the presence of fewer unique P2-HTX mutations in HNSCC-1, a clinical responder, versus the high number of unique P2-HTX mutations in HNSCC-2 and HNSCC-3 tumours, clinically classified as partial and non-responders, respectively, could indicate a propensity for the acquisition of new mutations and/or rearrangements during tumour propagation, consistent with the genetic instability. Furthermore, the ‘take rate’ in the current studies was <50%, consistent with published reports, which together with the long time required to establish a graft has been a limiting factor for translation of xenotransplant of primary models for predictive studies^58^. The *ex vivo* to *in vivo* functional correlation data clearly show the benefit of using CANScript technology as a surrogate of animal modelling. In addition, the minimal amount of tissue required to establish the CANScripts means multiple explants per tumour biopsy, which allows us to better capture the impact of intratumoral heterogeneity on outcome.

A powerful feature of the CANScript platform is its use of a novel machine learning approach that is tailored to make accurate predictions particularly for potential responders. Specifically, the algorithm operates in two stages: it first employs the recently proposed SVMpAUC-based learning algorithm to distinguish between responders and non-responders in a way that maximizes sensitivity (fraction of responders predicted as responders). Indeed, the learned model in our case achieved 100% sensitivity on the test set while keeping specificity in an acceptably high range. In the second stage, the algorithm learns an additional threshold to separate responder predictions into complete responder and partial responder predictions. This approach was found to be superior to the performance of a standard, widely used support vector ordinal regression algorithm that directly aims to make predictions in the three categories and does not explicitly incorporate the need for high sensitivity. Interestingly, studies have correlated complete pathological response to the long-term progression-free survival^59^, while recent ongoing clinical trials like adjuvant dynamic marker adjusted personalized therapy trial (ADAPT) are using short-term dynamic response prediction biomarkers like decrease in Ki-67 in clinical settings as surrogates for clinical outcome for tailoring personalized treatment^60^, indicating that integrating multiple end points into a single score as adopted in this model could make response prediction more comprehensive. Combined together, the comprehensive tumour ecosystem and the SVMpAUC-based algorithm makes the CANScript platform a powerful predictive tool that can be used across different tumour types and treatment regimens, as is evident from the overall response rates observed in HNSCC and CRC tumours to targeted and chemotherapy regimens that was similar to clinical outcome observed in previous studies^6^,^61^. Moreover, while for this study we have focused on predicting the patient response to a single drug regimen at a time, in the future, the approach can be extended to predicting a rank order among different drug regimens based on their likely outcomes, which could help in prioritizing different treatments. Furthermore, the CANScript platform can afford nearly high-throughput testing while capturing the patient intratumoral heterogeneity at a global level with higher fidelity, allowing predictions to be made within 7 days for truly personalizing chemotherapy.

## Methods

### Collection of tumour samples and patient sera

Tumours samples were collected by core biopsy at the beginning of treatment and at the time of surgical removal for deserving patients (for patient detail see Supplementary Methods). For each patient 5–10 ml of non-heparinized blood was collected at the time of first biopsy in BD-Vacutainer tubes and serum was separated at 1,000*g* for 15 min. All sera samples were aliquoted and stored at −80 °C for further use.

### Isolation of extracellular matrix proteins

Surgically removed fresh tumour tissues were dissected into small sections (~1–2 mm^3^) and digested with dispase (Stem cell Technologies Inc. ), and subsequently the cells were separated using a sieve^62^. Decellularization process was verified by phase contrast microscopy, and further confirmed by 4,6-diamidino-2-phenylindole staining and DNA quantification. Tissue slices suspended in dispase solution was incubated for 15 min at 48 °C. The tissues were homogenized in a high salt buffer solution containing 0.05 M Tris pH 7.4, 3.4 M sodium chloride, 4 mM of EDTA, 2 mM of N-ethylmaleimide and protease (Roche.11836153001) and phosphatase inhibitors (Sigma-aldrich, P0044 and P5726) using tissue homogenizer (Cole Parmer). The homogenized mixture was centrifuged repeatedly three times at 7,000*g* for 15 min and the supernatant was discarded to retain the pellet. The pellet was incubated in 2 M Urea buffer (0.15 M sodium chloride and 0.05 M Tris pH 7.4) and stirred for 1 h at 50 °C. The complex extracted proteins were solubilised in Urea buffer^63^. The mixture was then finally centrifuged at 14,000*g* for 20 min and resuspended in the 2 M Urea buffer, aliquoted and stored at −80 °C. In addition, extracted protein samples were run at denaturing conditions in the presence of standard molecular weight ladder. When the run was complete, the gel was transferred into a suitable staining tray and fixed in a solution containing formaldehyde in a shaker for 2 h. The gel was washed three times with 1 × wash solution once in every 5 min. The gel was incubated with sensitizing solution containing sodium-thiosulphate for 2 min with gentle shaking and visualized using silver staining.

### Identification of TMP components by nano LCMS/MS

The protein mixture was dissolved at the concentration of 1 μg ml^−1^ in 50 mM ammonium bicarbonate buffer. The pH of protein samples was adjusted to ~8.5. The samples (50 μl) were reduced with 10 mM DTT at 56 °C for 45 min, incubated at 95 °C for 5 min and then allowed to cool. Alkylation was carried out by using 55 mM final concentration of iodoacetamide in the dark. Trypsin (13 ng μl^−1^) was added at a ratio of 1:30 and enzyme/sample mixture was mixed well. Tubes with sample were placed into thermostat and incubated at 55 °C for 2 h and then 37 °C overnight. Digested samples were subjected for SpeedVac at 30 °C for 2–3 h. 5% formic acid was added for adjusting the pH to 3. The samples were either subjected directly to MS analysis or stored at −20 °C.

Sample was vacuum dried and reconstituted in 12 μl of 0.1% formic acid containing 12.5 fmol μl^−1^ bovine serum albumin (BSA) or β-gal. One micro liter of this was injected on column. Hence, the area of BSA/ β-gal was used for normalization. A separate Mascot run was performed with carboxymethylation as a dynamic modification to obtain area of BSA or β-gal protein. Area of the respective protein is normalized to the control area of respective sample. Reserpine (Sigma-aldrich) was used as a working standard. Digested peptides were subjected to analysis by injecting into nano LCMS/MS^63^. The instrument (STAR Elite, Q-TOF LCMS, Applied Biosystems) was externally calibrated with standard compounds. In brief, peptide mixtures were dissolved in 25 μl of sample preparation solution and injected (10 μl pick up) into nano-LC through an auto-sampler system. Peptides were eluted using nano-reverse phase column (Michrom C18 5 μm particle, 300 Å pore size, 75 μm ID, 150 mm length) which was further connected to the Nano Spray ESI-Q-TOF system (Qstar Elite, Applied Biosystems). A gradient of water and Acetonitrile was set up for 60 min with a flow rate of 400 nl min^−1^. Eluted peptides from the column were ionized using ESI source with ion spray voltage 2250 V and temperature 120 °C. Ionized peptides were analysed by one full MS scan and four consecutive product ion scans of the four most intense peaks, using rolling collision energy. An Information Dependant Acquisition (IDA) experiment was used to specify the criteria for selecting each parent ion for fragmentation, which included selection of ions in m/z range: >400 and <1600, of charge state of +2 to +5, exclusion of former target ions for 30 s, accumulation time of 1 s for a full scan and 2 s for MS/MS. The data generated by the Analyst software were stored in a.wiff format. The machine generated data files were analysed using ProteinPilot version 4.0 software with a combined NCBI Human Database (release 45, containing 39125 non-redundant protein entries, 18.8 Mb), Paragon Algorithm and Proteome Discoverer1.3 software. All searches were performed with tryptic specificity allowing two missed cleavages. Trypsin and keratin entries were retained in the list generated. During the analysis, in the search parameters modification of cysteine by idoacetamide and biological modifications programmed in algorithm were allowed. Mass tolerance for precursor ion and fragment ions were set to 100 p.p.m. and 0.2 Da, respectively. In Paragon Algorithm, protein score was calculated on the basis of percentage confidence level of the peptides identified. Protein score of minimum 0.47 (fit incorrect rate is 0%) corresponding to a confidence level >66% were used. To rule out false discoveries, we carried out a False Discovery Rate (FDR) analysis^64^ using ProteinPilot 4.0 with Paragon algorithm for data analysis. As part of the Paragon analysis method, a FDR analysis of the results was carried out by the Proteomics Performance Evaluation Pipeline Software (PSPEP). Finally, proteins were selected on the basis of their critical FDR value, that is, 1%. To avoid identifications based on redundant peptides in our proteome, we did not include proteins that have no unique peptide identifications. Protein grouping function was disabled for generation of protein list. Proteins that share some peptides as well as have unique peptide identifications were grouped accordingly. Deeper annotations were done by accessing specific published information.

For peptide and protein identification, peak lists were correlated with the human protein database^65^,^66^,^67^,^68^. The rationale for spectral counting derived protein abundance is that proteins in higher abundance result in more proteolytic peptides detected by tandem MS and subsequently identified by database searching. Following the matching of peptide peaks, peptide abundances in each of the analysed gradient fractions were calculated from the area under the peak. All data processing steps were manually inspected to ensure correct peak detection and matching; overlapping peaks were discarded. Proteins were considered quantifiable if they were represented in at least 75% of the clinical samples matching the cancer type and grade. There are many inherent variables, like ionization efficiency, sensitivity to digestion and interference at the time of elution might influence in the determination of the relative abundance for a protein. In general the prediction falls within a ratio of twofold compared with the actual one. Both sample distance and protein feature distance were calculated using Pearson’s correlation and average linkage was used for the clustering of both samples and protein features.

### Preparation, coating and detection of TMP mix

TMP cocktails were prepared, based on the relative abundance of key components obtained from LCMS/MS analysis of HNSCC and CRC patient tumour tissues using human proteins as shown in Supplementary Figs 2 and 3. Sterile culture wells were freshly coated with TMP cocktails (100 μg ml^−1^) unless mentioned otherwise. To visualize the coat, the matrix was incubated with anti-Collagen1 antibody at a dilution of 1:50 (rabbit polyclonal, Abcam. ab34710) for 1 h at room temperature. After four washes in PBS, slides were incubated with Alexa Fluor 555 (anti-mouse, Cell Signaling Technology.4409) for additional 45 min at room temperature in dark. Slides were washed with PBS and finally mounted with Vecta-Shield DAPI (Vector laboratories. H-1200) to confirm the absence of nuclear contamination in premixed TMP cocktail. Images were visualized under immunofluorescence microscopy setting using red and blue filters (DM4000, Leica Microsystems) and images were captured with DFC 425C (Leica) camera.

### Surface scanning electron microscopy

Electron microscope compatible cover slips (Thermanox, Ted Pella Inc.) were coated with freshly prepared TMP cocktails (100 μg ml^1^) for 4 h, washed twice and were fixed in 10% buffered formalin for 10 min (to resist metal coating and high electron beam), washed in PBS and dehydrated with 70 and 100% ethanol for 5 min each. Immediately before imaging, the slides were coated with gold and the images were captured using a Cambridge scanning electron microscope with EDAX attachment.

### Human tumour explant culture

Tumour tissues were sectioned into ~300 μm slices using McIlwain tissue chopper (TedPella). These tumour sections were randomized and cultured in 48-well flat bottom plates coated with stage and grade-matched TMP with RPMI medium supplemented with 2% AS, 8% FBS (Life Technologies. 10270-106), 1 × Insulin-Transferrin-Selenium (ITS, Life Technologies. 41400-045), 1 × GlutaMAX (Life Technologies. 35050-061) and 1 × penicillin, streptomycin and amphotericin B (Life Technologies. 15140-122). Tumour slices (*n*=3) were treated with either anti-EGF neutralizing antibody (rabbit monoclonal, clone D8A1, Cell Signaling Technology. 12157) or with TPF (for HNSCC) or with cetuximab+FOLFIRI (for CRC) or with dimethylsulphoxide (DMSO; vehicle control) for 72 h. The final concentration of DMSO was kept ≥0.01%. Media with drugs were changed every 24-h interval. A portion of each tumour slice was used for cell viability (assessed by WST) and remaining tumours were fixed in 10% buffered formalin and embedded in paraffin. The paraffin-embedded tumours were used for histological (hematoxylin and eosin stain) and IHC analysis including proliferation and cell death.

### Human tumour xenografts

Freshly isolated primary human tumours were washed in normal saline and cut into a small pieces (~5 mm^3^) and implanted subcutaneously onto the flanks (both sides) in immune compromised 5–6 weeks old, female severe combined immunodeficiency (C.B-17/IcrHsd-PrkdcscidLystbg, Harlan) mice. Tumour bearing mice (at the time of commencement of treatment maximum tumour size was restricted to 100–150 mm^3^) were treated with vehicle (0.9% normal saline;) or concurrent regimen of TPF (cisplatin 2.5 mg kg^−1^ body weight, docetaxel 20 mg kg^−1^ and 5Fu 50 mg kg^−1^) or single agent cetuximab (4 mg kg^−1^) for 3–4 weeks. Tumour volume was calculated using the following formula, Tumour volume (mm^3^)=(π/6) LWH; where L=length (mm), W=width (mm) and H=height (mm). All mice studies and experimental protocols were approved by the institutional animal ethics committee.

### Gene expression, exome and mutational analysis

See Supplementary Methods for details. The data are publically available at Gene Expression Omnibus through GEO series accession number GSE63544 and GSE63545; Biosample accession numbers, SAMN03271711, SAMN03271712 and SAMN03271713.

### Machine learning algorithm

We learned a model for predicting patient responses as NR/PR/CR in two stages. At the first stage, PR and CR labels were grouped together into a single responder (R) category, and the recently proposed SVMpAUC algorithm^69^ was trained on the training set of 109 patients to learn a model to assign the scores and predict NR/R for new test cases. Specifically, given a training set containing n examples (*x*_i_,*y*_i_), i=1,...,*n* (here *n*=109), where *x*_i_ is a feature vector containing the four functional read-outs for the i-th patient and y_i_ is 1 if the i-th patient is a responder and −1 otherwise, the SVMpAUC algorithm learns a weight vector **w** maximizing (a concave lower bound on) the partial area under the ROC curve (partial AUC) up to a specified false positive rate *β* (here *β*=0.25), defined as follows^70^


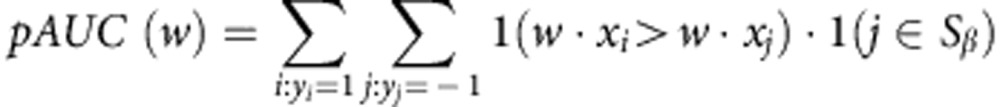


Where *S*_*β*_ contains indices j of the top β fraction of non-responders in the training set, ranked according to scores *w*.*x*_j_. This produced a weight vector *w* assigning coefficients of 0.2977, 0.5562, 0.0073 and 0.1388 to the viability, histology, proliferation and apoptosis read-outs, respectively. Together with a threshold of 19.1 corresponding to (approximately) *β*=0.25 false positive rate on the training set, this yielded an initial NR/R prediction model. In the second stage, the above model was further refined to classify the predicted responders as PR and CR; this was done by selecting a threshold (55.14) that maximized PR/CR classification accuracy on the training set.

### Statistical analysis

One way analysis of variance and Student’s *t-*test, linear regression and Spearman coefficient of correlation was analysed using GraphPad Prism version 5 for Windows, GraphPad Software.

## Author contributions

B.M., P.R., S.A., S.S. and P.K.M. were involved in the design of experiments. B.M., B.U, S.T., M.D., N.B., D.D.P. B.U.S., A.T. conducted *ex vivo*, *in vivo*, microarray, IHC and LCMS experiments and analysed the data. A.P. analysed the assay data. N.H. and S.A. were involved in algorithm development. B.M., S.T., P.R., M.S., S.A., S.S., P.M. wrote the manuscript. R.S., G.K.B., A.M.S., M.A.K. contributed to the clinical aspects of the study. G.B., P.H. conducted exome experiments and data analyses. R.B. and M.L. provided critical inputs for exome and pathology data and results. P.K.M. supervised the study.

## Additional information

**How to cite this article:** Majumder, B. *et al.* Predicting clinical response to anticancer drugs using an *ex vivo* platform that captures tumour heterogeneity. *Nat. Commun.* 6:6169 doi: 10.1038/ncomms7169 (2014).

## Supplementary Material

Supplementary Information Supplementary Figures 1-10, Supplementary Tables 1-3, Supplementary Methods and Supplementary References
