# Supplementary material for: Predicting clinical response to anticancer drugs using an ex vivo platform that captures tumour heterogeneity
Source: Nat Commun. 2015 Feb 27;6:6169. doi: 10.1038/ncomms7169 (PMC4351621; doi:10.1038/ncomms7169)
Supplement: Supplementary Information — Supplementary Figures 1-10, Supplementary Tables 1-3, Supplementary Methods and Supplementary References [file ncomms7169-s1.pdf]

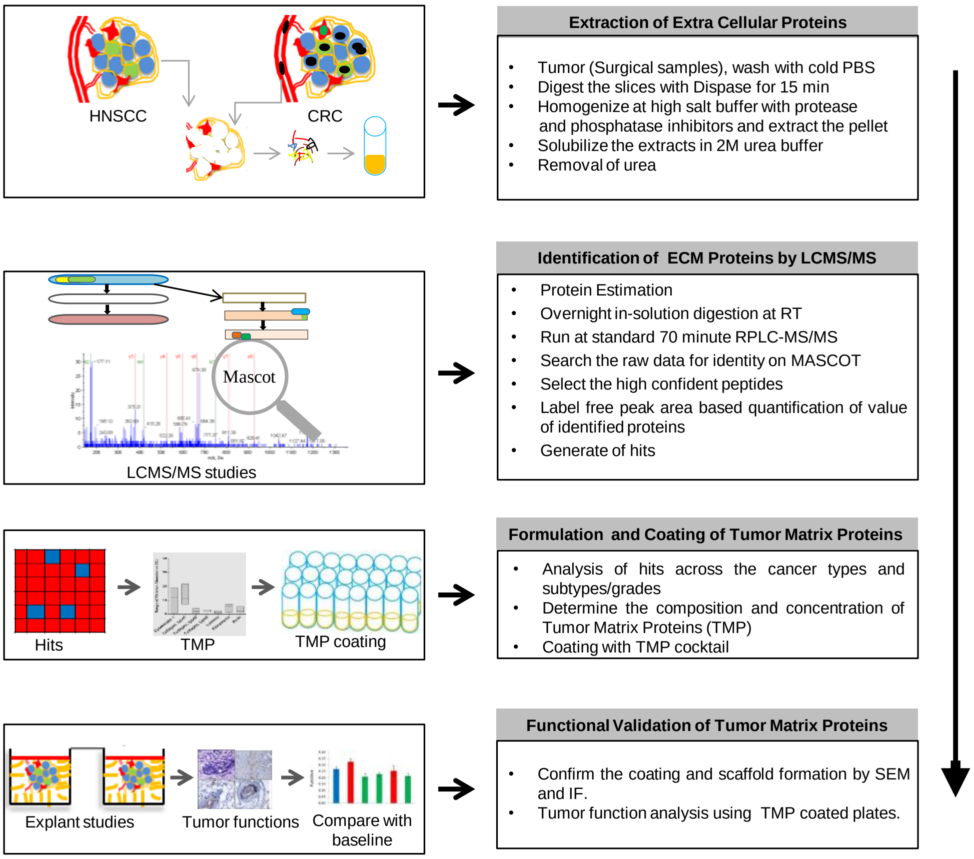

Supplementary Figure 1. Work flow for identification, formulation and validation of Tumor Matrix Proteins (TMP) from HNSCC and CRC specimens.

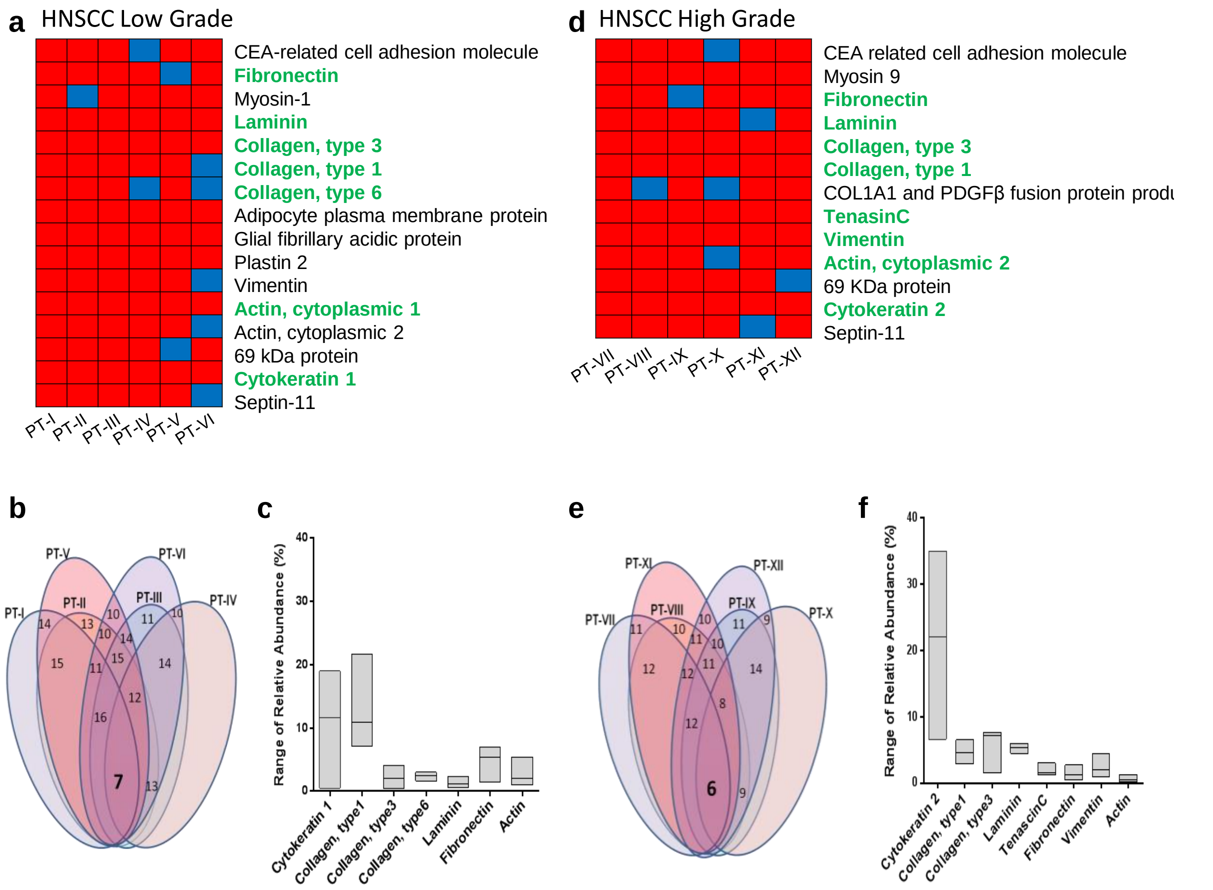

Supplementary Figure 2. Identification of key matrix protein components by LCMS/MS for HNSCC. LCMS/MS analysis of lower (low pathological grade, 1/2;) and higher grade (high pathological grade, 3/4;) of HNSCC tumor specimens revealed distribution of common and unique proteins for a given grade. (**a**) Heat map was generated from LCMS data obtained from lower grade HNSCC tumors (n=6). At the time of protein selection, FDR was kept as minimal as 1%. The color code describes the presence (Red) and absence (Blue) of corresponding common proteins listed. Proteins in bold were being selected for the preparation of TMP cocktail and used for functional validation of HNSCC lower grade tumors. Bottom of the map indicates corresponding patient ID. (**b**) Venn Diagram shows number of overlapped matrix proteins identified between different tumors. (**c**) Box plot refers to the range of relative abundance of key TMP identified and subsequently customized for coating HSNCC low grade tumor specimens. Horizontal line in the box represents median **(d)** Heat map was generated from the LCMS data obtained from HNSCC higher grade tumors (n=6). At the time of protein selection, FDR was kept as minimal as 1%. The color code describes the presence (Red) and absence (Blue) of corresponding common proteins listed. Proteins in bold were being selected for the preparation of TMP cocktail for functional validation of HNSCC higher grade tumors. (**e**) Venn Diagram shows number of overlapped matrix proteins identified between the samples. (**f**) Box plot refers to the range of relative abundance of TMP identified and subsequently customized for coating of HSNCC high grade tumor specimens. Horizontal line in the box represents median

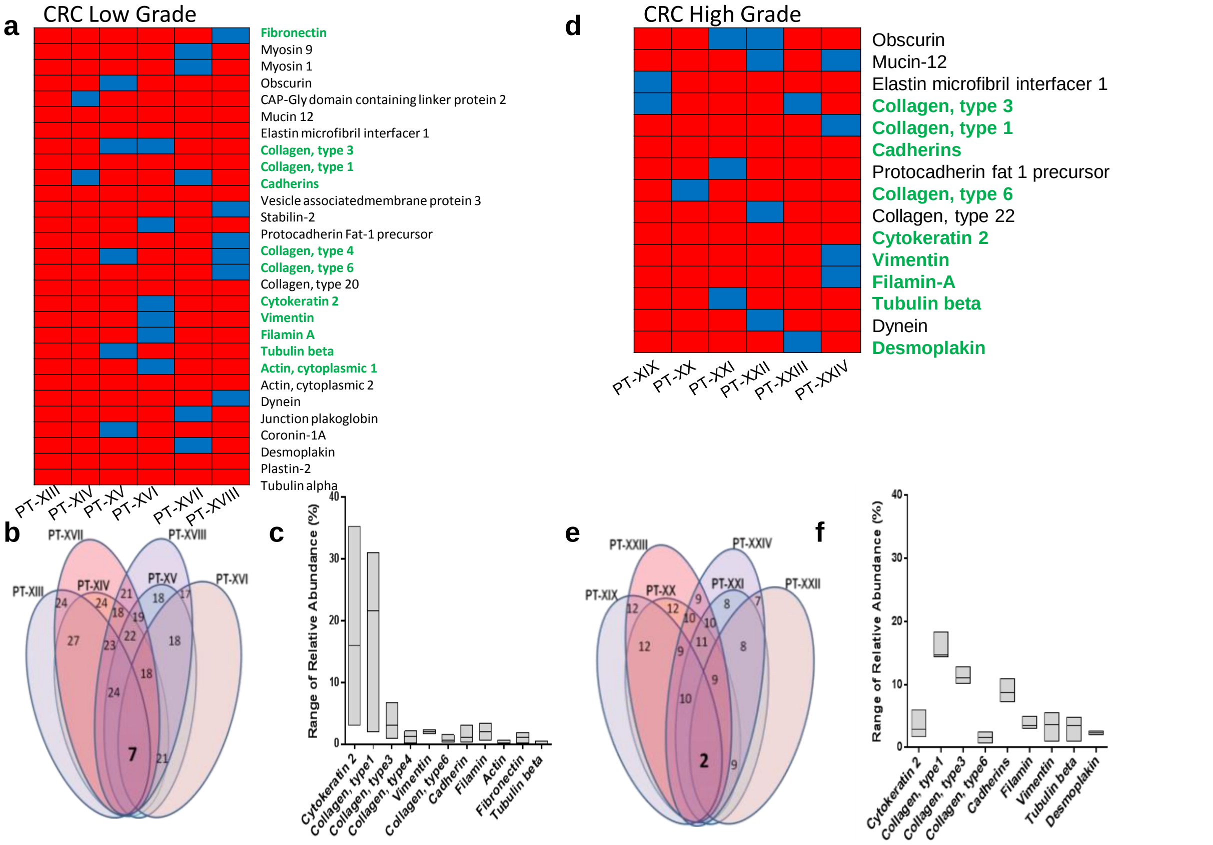

Supplementary Figure 3. Identification of key matrix proteins components by LCMS/MS for CRC. LCMS/MS analysis of lower (low pathological grade, 1/2) and higher grade (high pathological grade, 3/4;) of CRC tumor specimens revealed distribution of common and unique proteins for a given grade. (**a**) Heat map was generated from the LCMS data from CRC lower grade tumors (n=6). At the time of protein selection, FDR was kept as minimal as 1%. The color code describes the presence (Red) and absence (Blue) of corresponding common proteins listed. Proteins in bold were being selected for the preparation of TMP cocktail and used for functional validation of CRC lower grade tumors. Bottom level indicates individual patient ID (**b**) Venn diagram shows number of overlapped matrix proteins identified between the samples of low grade. Center bold number indicates protein common for all 6 samples. (**c**) Box plot refers to the range of relative abundance of TMP identified and subsequently customized for coating of CRC low grade tumor specimens. Horizontal line in the box represents median (**d**) Heat map was generated from LCMS data from CRC higher grade tumors (n=6). At the time of protein selection, FDR was kept as minimal as 1%. The color code describes the presence (Red) and absence (Blue) of corresponding common proteins listed. Proteins in bold were being selected for the preparation of TMP cocktail and used for functional validation of CRC higher grade tumors. (**e**) Venn Diagram shows number of overlapped matrix proteins identified between the samples of high grade. (**f**) Box plot refers to the range of relative abundance of TMP identified and subsequently customized for coating of CRC high grade tumor specimens.

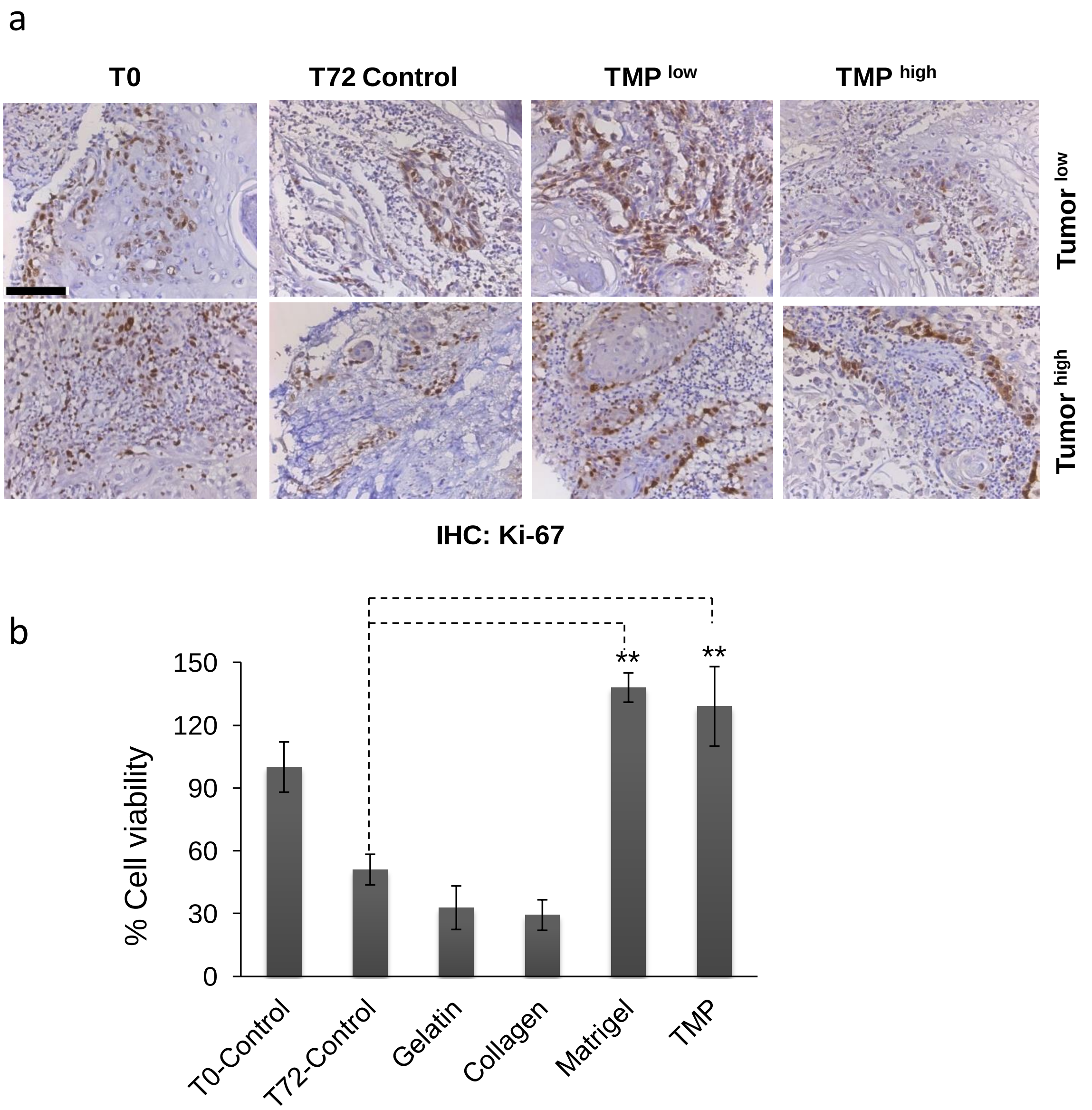

Supplementary Figure 4. Effects of low and high grade TMP cocktails for matched and unmatched tumors and comparison with commercially available matrix support. (**a**) Primary HNSCC tumors of lower (top row) and high grade (bottom row) were cultured for 72 hours in tissue culture plates coated with no TMP (column 2), low grade TMP (column 3) and high grade TMP (column 4). The tissue slices were embedded in paraffin and stained for Ki-67 and expression under different coating conditions were compared with baseline tumors (T0 control, 1st column). (**b**) Graph shows percent cell viability in triplicates for various coating conditions as indicated (mean ± s.d) for HNSCC. ***P* < 0.01 compared to T72 control (ANOVA, *n*=8).

a

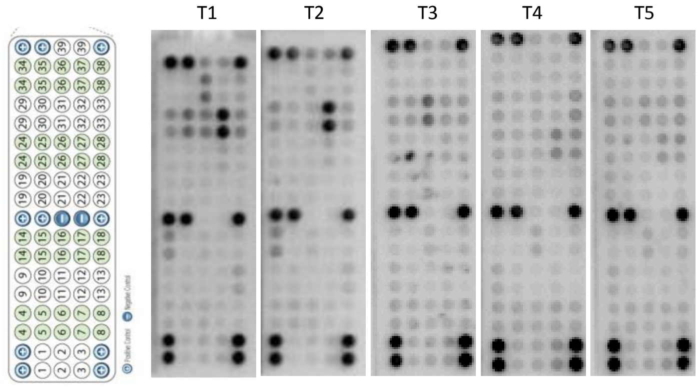

b

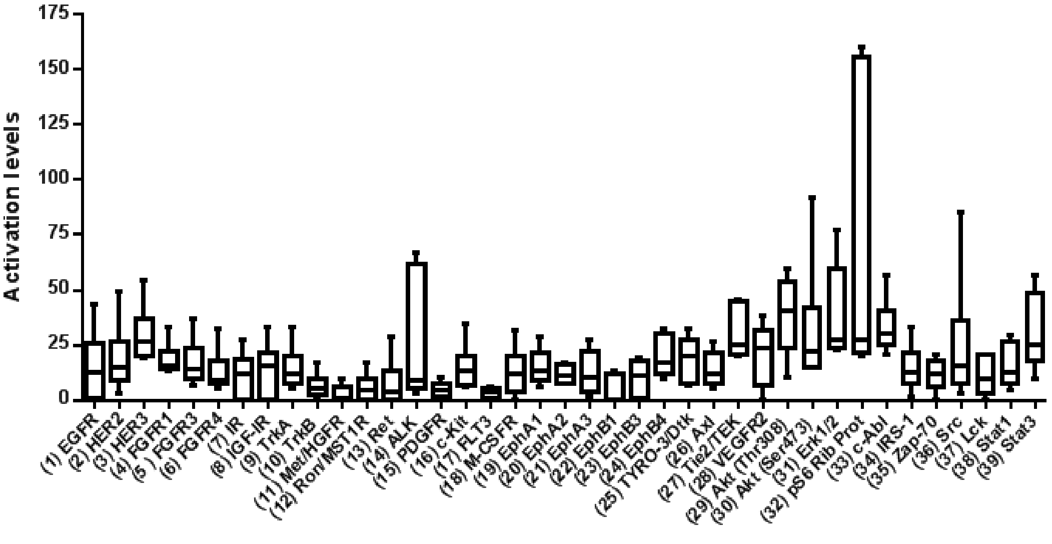

Supplementary Figure 5. Reverse phase phosphoproteomic profiling of tumor specimens from HNSCC patients. (**a**) Array Target Map (left) for RPPA (Cell Signaling Technology, #7982). Total cell lysates were applied to slides pre-coated with different antibodies against RTK pathways. Signal was detected by chemiluminiscence method and spot intensities were observed for five representative patient tumors (T1, T2, T3, T4 and T5). (**b**) Expression of RTKs (*n*=5). Quantification of RTK activation was performed by measuring the signal intensity of individual analytes normalized to negative control. Box whisker plot represents the range of activation. Horizontal line represents the median, the bottom and top of the box represent first and third quartile (25 and 75 percentile) respectively, error bars (wiskers) represent the interquartile range.

.

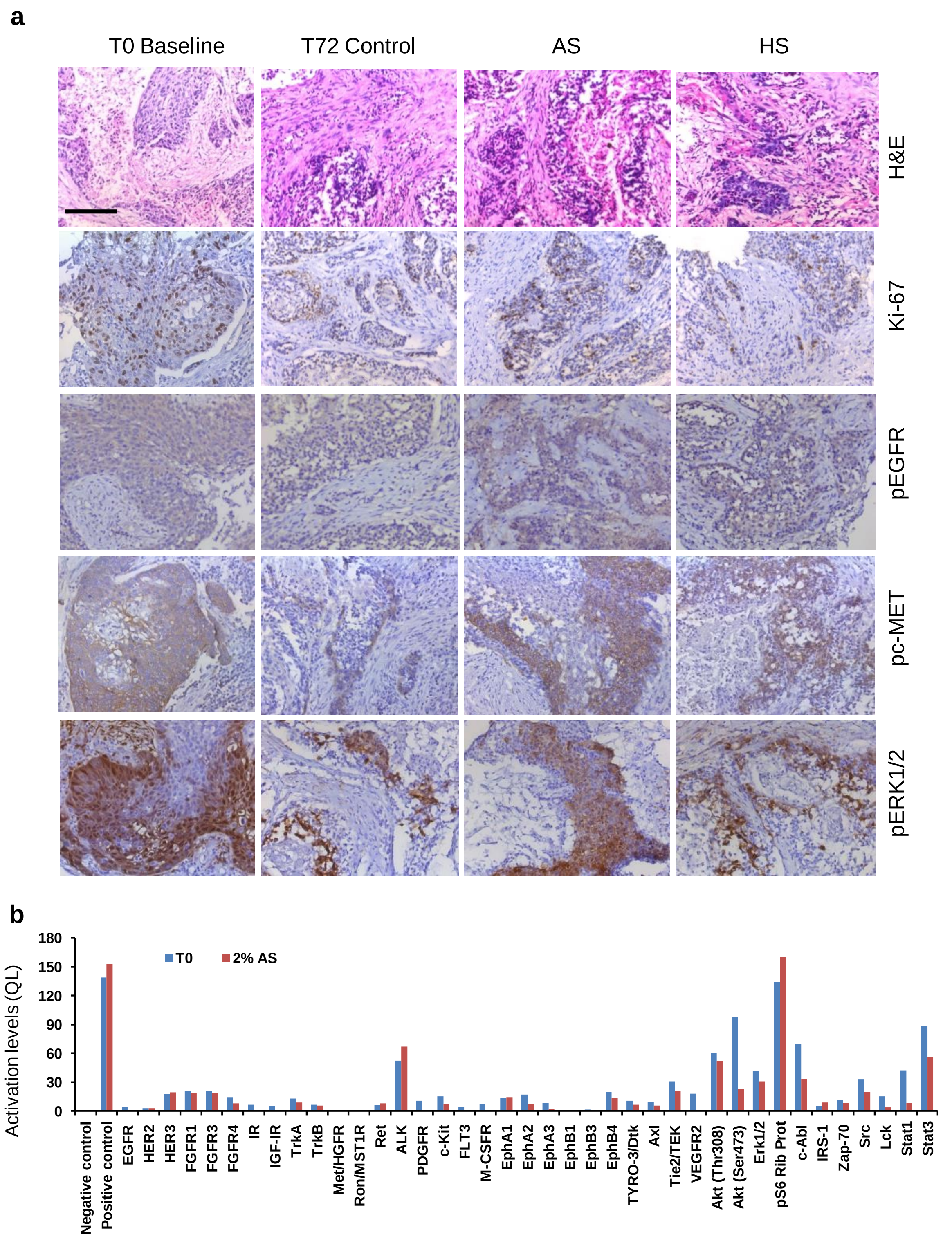

Supplementary Figure 6. Activation of key signaling molecules with autologous ligands conserves the base line signaling as compared with heterologous ligands. (**a**) Tumor explants were treated with FBS alone (72 hours Control), 2% AS (AS) and 2% HS (HS) for 72 hours and stained with H&E, Ki-67, phospho-EGFR, phospho-MET, and phospho-ERK1/2. Representative stained images were shown. Scale bars indicate 50 µm. (**b**) Quantification of comparative expression levels of RTK analytes at T_0 baseline and 48 hours post culture with 2% AS by measuring the signal intensity of individual analytes using spot analysis software.

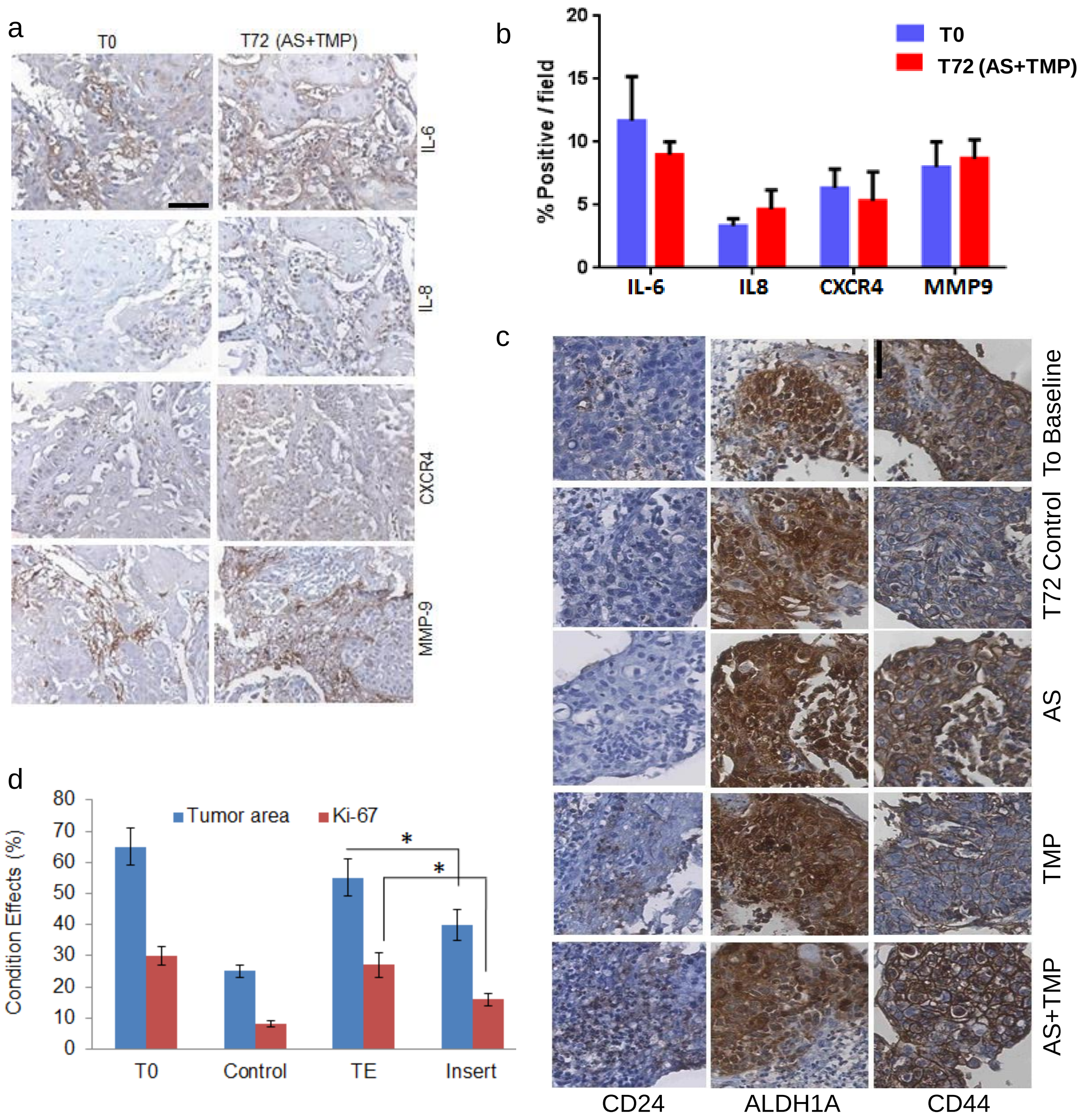

Supplementary Figure 7. Markers associated with stroma and tumor initiating cells are retained over 72 h when cultured in CANScript ecosystem. .Primary HNSCC samples were cultured in tissue culture plates in presence of 8% FBS (T72 control), 2% AS alone (T72AS), TMP alone (T72 TMP), combination of TMP and AS (T72 AS+TMP). Baseline tumors (T0 control) and tumors cultured for 72 h were embedded in paraffin. (**a**) IHC profile of IL-6, IL-8, CXCR-4 and MMP-9 after 72 hours of culture. Scale bar indicates 100 µm. (**b**) Corresponding IHC scores showing percent positive area per field for each indicated marker in triplicates (*n*=*5*). (**c**) Markers associated with cancer stem cell after 72 hours in CANScript explants were evaluated by IHC using antibodies against CD44, CD24 and ALDH1A. Scale bar indicates 50 µm. (**d**) Graphical representation of tumor morphology and proliferation comparing CANScript and organotypic insert. HNSCC tissues were cultured in plates coated with TMP along with 2% AS, (TE explant) and without TMP and AS (T72 control). In parallel, tumors obtained from same patients were cultured in wells equipped with organotypic inserts. Baseline tumors (T0 control) and tumors cultured for 72 h were evaluated for head to head comparison of Ki-67 and overall tissue morphology. All data indicate mean ± s.d. in triplicates (n=9). *$P \leq 0.01$ compared to corresponding T72 no treatment control (by ANOVA).

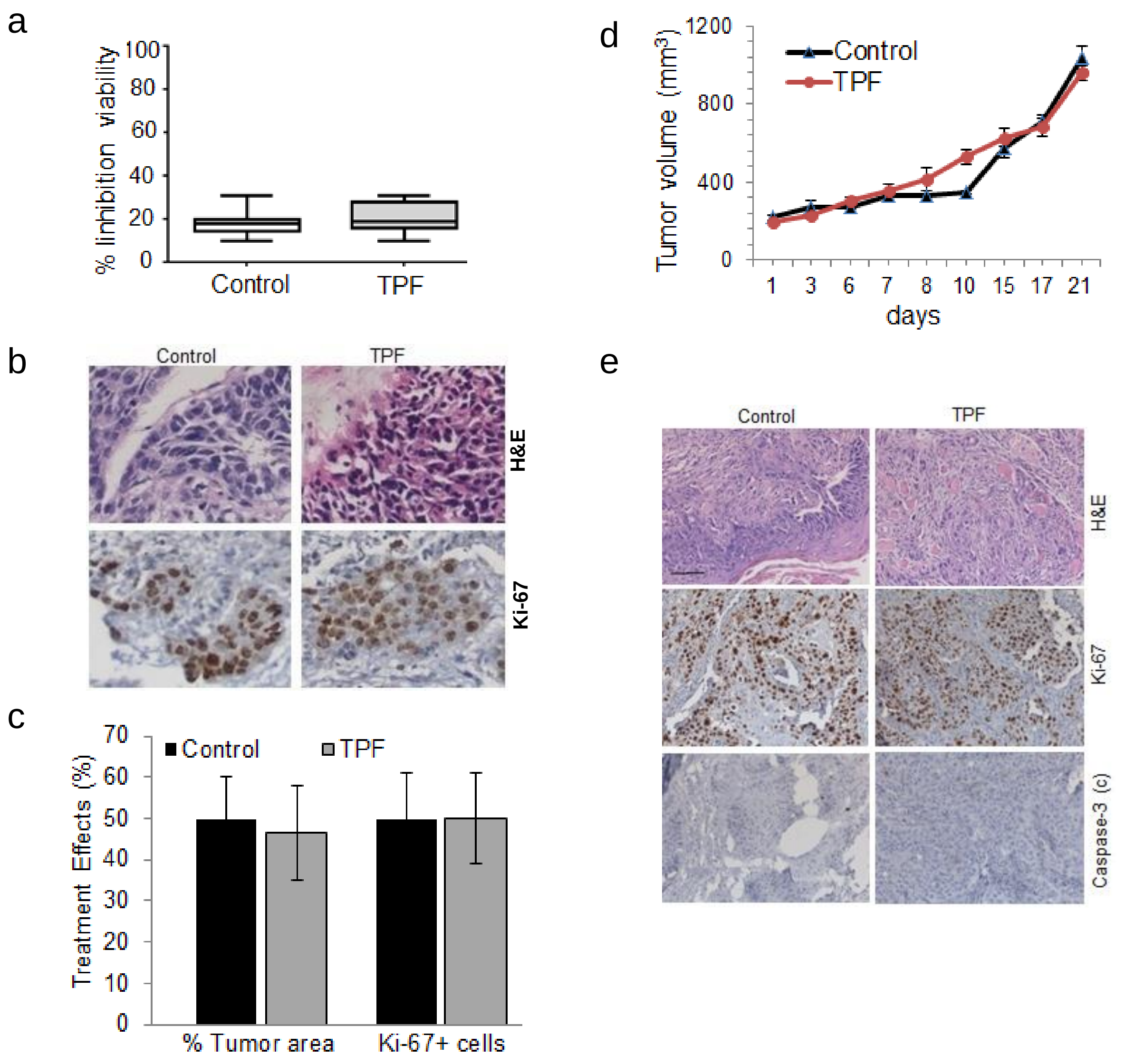

Supplementary Figure 8. HNSCC tumors predicted to be TPF non-responders do not exhibit antitumor response in a matched HTX mouse model (**a**) Biopsied tumors from HNSCC HTXs were sectioned (~300 µm) and cultured in TMP coated 48 well plates with 2% AS and 8% FBS in RPMI media for three days with DMSO (Control) or TPF. Cell viability was measured by WST and percent inhibition of cell viability was determined using T_0 value. Box plot shows viability in TPF-treated tumors compared to control in multiple donors (*n*=9). Horizontal line in the box represents the median, error bars represent the interquartile range (**b**) Corresponding IHC profile. Tumor sections treated with DMSO (control) and TPF were stained with H&E and antibodies against Ki-67. Scale bar indicates 50 µm. (**c**) Graph shows percentage of tumor area and number of Ki-67+ cells per sections from control and TPF treated explants. All data are represented as mean of triplicates ± s.d. (**d**) HNSCC patient tumors (which did not respond to TPF in explants setting) derived mice were treated daily with normal saline (Control) or TPF for 21 days. Tumor volumes were measured at indicated time points. Data are mean tumor volume ± s.d. of 6 mice per group. (**e**) Representative IHC features of tumors at the end of treatment. Tumors dissected from euthanized mice from both control and TPF groups were embedded, sectioned and stained with H&E, anti-Ki-67 antibodies and caspase-3 (active) as indicated. Scale bar is 100 µm.

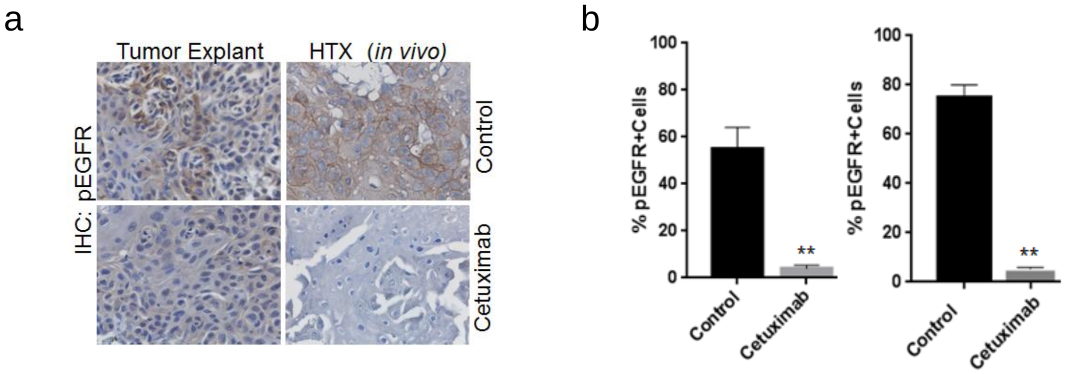

Supplementary Figure 9. Inhibition of target in HNSCC tumors treated with cetuximab in *ex vivo* and *in vivo* settings (**a**) Cetuximab-mediated inhibition of target in HNSCC was determined by IHC using anti phospho-EGFR antibody in both CANScript explant (left) and *in vivo* HTX settings (right). (**b**) Corresponding IHC scores from CANScript and *in vivo* HTX represented as the mean percent of pEGFR+ve cells per section in triplicates following exposure to control or cetuximab for 8 h. All data indicate mean ± s.d. in triplicates. ***P*≤0.01 compared to no treatment control by paired *t*--test.

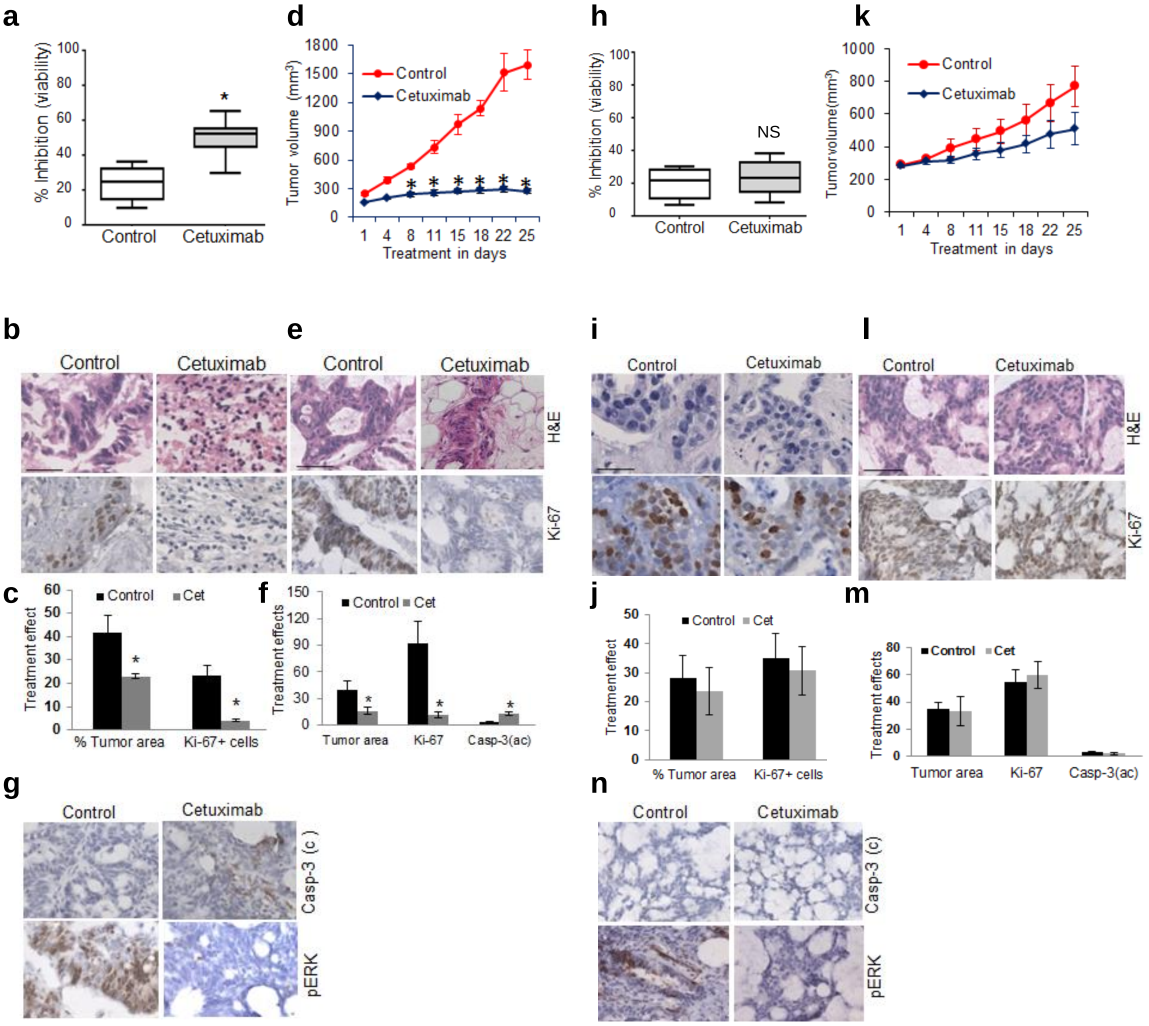

Supplementary Figure 10. CANScript and HTX *in vivo* correlation in CRC. Tumors from CRC patient were sectioned (~300 μm) and cultured in TMP-coated wells with 2% AS and 8% FBS in DMEM media for three days with DMSO (Control) and cetuximab. (**a**) Cell viability was measured at 72 h post treatment. Box plot represents percent inhibition of cell viability in cetuximab-treated tumors in multiple experiments compared to corresponding controls (*n*=9). ***P*<0.001 compared to control as calculated by *t*-test. Horizontal line in box represents the median, error bars represent the interquartile range.(**b**) Representative histology and IHC profiles illustrate morphology and proliferation respectively. Tumor sections treated with DMSO (control) and cetuximab were stained with H&E and anti-Ki-67. Scale bar indicates 50 μm. (**c**) Percentage of tumor area and number of Ki-67+ cells per section from control and cetuximab treated explants. All data are represented as mean of triplicates ± s.d. **P*<0.001 compared to control (*t*-test). (**d**) Tumor growth inhibition in cetuximab-treated mice. CRC patient tumors that showed noticeable response to cetuximab in TE explant were maintained as HTX in mice and treated with normal saline (Control) or cetuximab three times a week for 25 days. Tumor volumes were measured at indicated time points. Data are mean tumor volume ± s.d. of 10 mice per groups. **P*<0.001 compared with corresponding vehicle control. (**e**) Representative IHC profile of tumors harvested from drug treated mice. Tumors were harvested from euthanized mice 6 h after the last dose of cetuximab. (**f**) Tumor sections were stained with H&E, anti Ki-67 antibodies. Scale bar is 50 μm. Graph shows quantification of the mean of percent tumor area, number of Ki-67+ cells and cleaved caspase-3-positive cells per section from vehicle control and cetuximab treated

mice. All data indicate mean ± s.d. of 6 mice per group. *P<0.001 compared to corresponding vehicle control. **(g)** Tumor sections were stained with, anti caspase-3 (active) and anti pERK1/2 antibodies. (**h**) Data show that CRC tumors non-responsive to cetuximab in CANScript platform also reflect similar (**k**) *in vivo* results. Cell viability was measured at 72 h post treatment. Box plot represents percent inhibition of cell viability in cetuximab-treated tumors (n=9). Horizontal line in the box represents the median, error bars (wiskers) represent the interquartile range. ^{NS}P<0.2 compared to control (n=9) as calculated by paired t-test. (**i**) Micrographs show representative IHC profile of proliferation and morphology. Scale bar is 50 µm. (**j**) Percentage of tumor area and number of Ki-67+ cells per sections from control and cetuximab treated explants. All data are represented as mean of triplicates ± s.d. (**k**) Graph shows *in vivo* efficacy of cetuximab using the same CANScript-predicted non-responder tumors. Tumor volumes were measured at indicated time points. Data are mean tumor volume ± s.d. of 10 mice per group. (**l**) Representative IHC profile of tumors harvested from drug treated mice matching with tumor inhibition *in vivo*. After 25 days of treatment, tumor sections were stained with H&E, anti Ki-67, cleaved caspase-3 and anti pERK1/2 antibodies. Scale bar indicates 50 µm. (**m**) Data represented as percent tumor area, number of Ki-67+ cells and cleaved caspase-3 positive cells per section from vehicle control and cetuximab treated mice. All data indicate mean ± s.d. of 6 mice per group. (**n**) Micrographs compare the level of cleaved caspase-3 and pERK in a non-responder post-treatment with cetuximab or vehicle. Scale bar indicates 50 µm.

Supplementary Table 1. Patient demographic details. cancer stages and tumor sites.

HNSCC	Number	%
Total	112	
**Age**		
Age >= 50	64	57
Age < 50	48	43
**Gender**		
Male	80	71
Female	32	29
**Cancer Stage**		
Stage IV	42	37
Stage III	36	32
Stage II	27	24
Stage I / unknown	7	6
**Tumor site**		
Buccal Mucosa	7	6
Others	32	29
Glottis	6	5
Larynx	16	16
Maxilla	4	3
Oral Cavity	4	3
Pharynx	20	18
Pyriform Fossa	4	3
Pyriform Sinus	11	10
Tongue	8	6

CRC	Number	%
Total	52	
**Age**		
Age >= 50	38	73
Age <50	14	27
**Gender**		
Male	30	58
Female	22	42
**Cancer Stage**		
Stage IV	30	58
Stage III	19	36
Stage II	2	4
Stage I / unknown	1	2
**Tumor site**		
Anorectal cancer	1	2
Colon Cancer (Metastases)	15	29
Colorectal	6	12
Rectal Cancer	9	18
Rectosigmoid colon carcinoma	8	16
Sigmoid Colon	12	23

Supplementary Table 2

Primers sequence and PCR conditions

Gene name	Primer sequence	Target length bp	Annealing temperature ^{o}C
PDGFA	F: 5'-CACACCTCCTCGCTGTAGTATTTA- 3'	220	55
	R: 5'-GTTATCGGTGTAAATGTCATCCAA- 3'		
DUSP1	F: 5'-TTTGAGGGTCACTACCAG-3'	248	55
	R: 5'-GAGATGATGCTTCGCC- 3'		
STAT3	F: 5'-ATCACGCCTTCTACAGACTGC-3'	176	55
	R: 5'-CATCCTGGAGATTCTCTACCACT-3'		
FABP4	F: 5'-AGCACCATAACCTTAGATGGGG-3'	132	50
	R: 5'-CGTGGAAGTGACGCCTTTCA-3'		
ITSN1	F: 5'-GGCAATTATTCAATAGTCAT-3'	126	55
	R: 5'-TGTCAGAAAGATTCCATAT-3'		

Supplementary Table 3

Exome sequencing analysis shows potential key genes deregulated in HNSCC

	**HNSCC 1**
PATHWAY	Pathway 1_2
PRC2_EDD_UP.V1_DN	Genes down-regulated in TIG3 cells (fibroblasts) upon knockdown of EED [GeneID=8726] gene. CDHR2, CUBN, GPC5, GSG1, HIVEP2, PCGF3.
MTOR_UP.N4.V1_DN	Genes upregulated in CEM-C1 cells (T-CLL) by everolimus and mTOR pathway inhibitor. CD1A, EFHC1, ZNF14.
TBK1.DF_DN	Genes down-regulated in epithelial lung cancer cell lines upon over-expression of an oncogenic form of KRAS [Gene ID=3845] gene and knockdown of TBK1 [Gene ID=29110] gene by RNAi. ALDH9A1, C15orf29, NEK1, NRIP1.

	**HNSCC 2**	**HNSCC 3**
PATHWAY	Pathway 3_4	Pathway 5_6
**GSEA_C2_KEGG pathway**		
KEGG_MAPK_SIGNALING_PATHWAY	CACNA1A, CACNG7, FGF23, FLNB, RASA2, RRAS2	CACNA1C, DAXX, FGFR2, IL1B, MAP3K1, MAP3K12, NF1, RASGRP3
KEGG_WNT_SIGNALING_PATHWAY	FZD6	FZD1, PLCB1, ROCK2, TCF7L2
KEGG_TGF_BETA_SIGNALING_PATHWAY	RBL1, SMAD1, THBS3	RBL2, ROCK2, THBS2
KEGG_AXON_GUIDANCE	ABLIM1, EPHA3, EPHB1, FYN, NTN1, PLXNA2, ROBO1, SEMA4F	ROCK2, UNC5C
KEGG_PATHWAYS_IN_CANCER	BAX, BIRC3, COL4A2, FGF23, FZD6, GLI2, GLI3, LAMA4, LAMB1, MSH2, MSH6, PIAS2, PIK3CG, STK36, VEGFC	FGFR2, FZD1, TCF7L2
KEGG_COLORECTAL_CANCER	BAX, MSH2, MSH6, PIK3CG	

Note:

In pair#1 (i.e. HNSCC-1 and its HTX) the candidate drivers were *AKT2* and *CASP8.*

In pair #2 (i.e. HNSCC-2 and its HTX) the key drivers appear to be *BCL9* and *ROS1*.

In pair#3 (i.e HNSCC-1 and its HTS) *JAK3* and *SYK* are putative candidate drivers.

Enriched pathway derived point mutations in primary and HTX pair identified genes largely involved in MAPK, TGF-□ and WNT signaling pathways at least in case of pair #2 and #3.

Additionally, *ABLIM1* (in pair #2) and *ROCK 2* (in pair #3) -both linked to axon guidance, pathways in cancer and colorectal cancer associated genes like *BAX, FGFR2* and *TCF7L2* were covered by both these pairs.

In contrast, pair #1 demonstrated predominantly shared mutation in genes linked to mTOR and oncogenic *KRAS* activation. In addition to previously identified genetic alterations (TP53, PTEN, NOTCH1) the novel mutations (*AKT2, ROCK, ROS, JAK3, SYK*) were found in primary tumors.

Supplementary Methods

Patient enrolment and treatment

Patients diagnosed with locally advanced Head and Neck Squamous Cell Carcinoma (HNSCC) and Colorectal Cancer (CRC), undergoing treatment at Mazumder-Shaw Cancer Center, Kidwai Memorial Institute of Oncology, Bangalore, India and Stanley Medical College, Chennai, India were included in this study. The exclusion criteria followed for the study include pre-existing infections such as HIV-1, HCV, HBV, and other disorders such as neurological problems, cardiovascular diseases, respiratory/renal failure etc as per clinician's guidelines. Otherwise no deviation was made in terms of treatment and care because of this study. Informed consent (in three languages including patient's mother tongue) was obtained from all patients recruited in this study. The study was approved by respective institutional review board (IRB). For HNSCC the standard initial dose of docetaxel, cisplatin, and 5-fluorouracil (TPF) was given to each patient based on currently accepted treatment protocol and clinician's guideline for a total of three cycles whereas CRC patients received cetuximab + FOLFIRI. Clinical as well as histological/radiological responses were assessed before and after the third cycle of treatment by a qualified pathologist and nuclear medicine experts. The classification of responders and non-responders was done based on clinical and radiological (PERCIST) criteria.

Reagents for LCMS

All solvents and reagents used were of LCMS quality. Trypsin preparation (i) for bulk use, 13 ng μl^{-1} of Trypsin (Trypsin Gold, LCMS Grade, Promega, Wisconsin, USA. V5111) was prepared in 10 mM ammonium bicarbonate (Sigma-aldrich, St Louis, USA. 40867) containing 10% (vol/vol) Acetonitrile. Twenty microgram of Trypsin from the vial was dissolved in 1.5 ml of the buffer. The stock has been prepared shortly before use and unused volume in general was discarded. (ii) For a small volume of Trypsin buffer, the 20 µg lyophilized enzyme was dissolved in 1.5 ml of 1 mM HCl (13 ng μl^{-1} Trypsin prepared) and 100 µl aliquots were stored at -20 °C. After thawing the frozen aliquots, pH was adjusted by adding 15 µl of 50 mM ammonium bicarbonate. Ammonium bicarbonate (50 mM, Mass Spec grade, Sigma-aldrich, St Louis, USA. 40867) solution was freshly made in large (50-100 ml) volume and discarded after use. 5% Formic acid was prepared (vol/vol) in water. 1 M Dithiothreitol (DTT, Molecular Biology grade, Sigma, St Louis, USA. 43815) stock was made in 50 mM Ammonium Bicarbonate. Iodoacetamide (IAA, Sigma-aldrich, St Louis, USA. I6125) was prepared in 10 mg ml-1 stock with ultrapure LCMS grade water. For protein estimation BioRad's DC protein assay kit (500-0116) was used. Sample preparation solution for LCMS was obtained at the ratio of 98% water, 2% Acetonitrile (Sigma-aldrich, St Louis, USA. 14261) and 0.5% Formic acid. Compositions of LC gradient solutions were described

as follows. Buffer 1A- Acetonitrile (2%), water (98%) and Formic acid (0.2%); Buffer 2A- Acetonitrile (2%), water (98%) and Formic acid (0.2%); Buffer 2B- Acetonitrile (98%), water (2%) and Formic acid (0.2%). Reserpine (Sigma-aldrich, St Louis, USA. 43530) was used as working Standard. BSA (Sigma-aldrich, St Louis, USA. 05470) and β galactosidase (Sigma-aldrich, St Louis, USA. G6008) were used for trypsin digestion along with samples.

ELISA

Serum was isolated from patient blood and the levels of EGF (Life Technologies. KHG0061), HGF (Life Technologies. KAC2211),VEGF (Abcam. ab100662) and MCSF (Abcam. Ab100590) were determined by Sandwich ELISA.

Reverse phase phosphoproteomic array

HNSCC tumors slices were cultured with 2% autologous serum for 48 h. Both original and cultured tumor tissues were lysed with 1x cell lysis buffer (Cell Signaling Technology. 9803). Total protein was estimated by modified Lowry method (Bio-Rad's DC Protein Assay) and phosphorylation status of 39 proteins was detected using PathScan RTK signaling antibody array kit (Cell Signaling Technology. 7982). Intensity of images was quantified using image analysis software.

Cell viability assay

Tumor cell viability was assessed by Cell Counting Kit-8 (CCK-8) (Dojindo. CK04-13). Briefly, one tenth volume of CCK-8 solution was added to each well containing tissue slices and incubated at 37 °C for 4 h in a CO_2 incubator under humidified condition. The absorbance was measured at 450 nm using a microplate reader (Bio-Rad). Cell viability was assessed with appropriate controls.

***In vitro* ATP utilization assay**

Thirty micro molar ATP was spiked to the explant culture and further incubated for 30 min at a standard culture condition. ATP level was determined using StayBrite ATP assay system (BioVision, K791-100). The ATP utilization was calculated by subtracting the ATP levels in the explant free control (blank) from treated and untreated explants groups.

Histopathological analysis

A part of tumor tissue was fixed in 10% buffered formalin and embedded in paraffin (FFPE). Five micron FFPE tissue sections were stained with hematoxylin and eosin (H&E). All tumors were graded according to standard pathological criteria. The tumor content and

tumor-stroma ratio of tissues was also assessed for developing histology score similar to method described previously[1].

Immunohistochemical (IHC) analysis

Formalin fixed paraffin embedded (FFPE) tissue sections were serially collected to maintain basic histological features. The tissue sections were de-paraffinized followed by rehydration. The sections were incubated with Antigen Unmasking Solution (Vector Laboratories. H-3300) for 10 min followed by retrieval using a microwave for 30 min. Endogenous hydrogen peroxidase was quenched by incubating the sections with 3% H_2O_2 (Merck. 61868505001730) for 15 min and washed in running tap water for 3 min followed by a wash in 1x PBS for 7 min. Protein blocking was performed using 10% goat serum (Vector Laboratories. 5-1000) for 1 h at room temperature. Tissue sections were incubated with following primary antibodies: anti human Ki-67 (rabbit polyclonal from Vector Laboratories, 1:600 dilution. VP-K451), anti human GLUT1 (1:200 dilution, rabbit polyclonal from Abcam. ab15309), anti human cleaved Caspase-3 (1:600 dilution, rabbit polyclonal, clone D175, from Cell Signaling Technology. 9661), anti human CD34 (mouse monoclonal, clone QBEnd/10. Biogenex. AM236-5M) and corresponding secondary antibody (SS Multilink, Biogenex. HK340-5K), anti human CD68 (mouse monoclonal, clone KP1, from Dako. IS609), anti human Vimentin (1:100 dilution, mouse monoclonal, clone Vim 3B4, from Dako. M7020), anti human E-Cadherin (1:200 dilution, rabbit monoclonal, clone 24E10, from Cell Signaling Technology. 3195), anti human IL-6, (1:400 dilution, rabbit polyclonal from Abcam. ab6672), anti human IL8 (1:50 dilution, rabbit polyclonal from Abcam. ab7747), anti human CXCR4 (1:100 dilution, rabbit polyclonal from Abcam. ab2074), anti human MMP-9 (1:100 dilution, rabbit monoclonal, clone EP1254, from Abcam. ab76003). Additionally antibodies against cancer stem cell (CSC) related markers namely CD44 (dilution 1:100, mouse monoclonal, clone 3C11, Cell Signaling Technology. 3570), CD24 (1:50 dilution, mouse monoclonal, clone ML5, BD Biosciences. 555426) and ALDH1 (1:250 dilution, mouse monoclonal, BD Biosciences. 611194) were also included. For all these antibodies incubation was carried out at RT for 1 h. Following phospho-specific antibodies were used: anti human phospho ERK1/2 (1:200 dilution, rabbit monoclonal, clone D13.14.4E, from Cell Signaling Technology. 4370), anti human phospho AKT (1:50 dilution, rabbit monoclonal, clone D9E, from Cell Signaling Technology. 4060) and anti human phospho EGFR-Tyr1068 (1:200 dilution, rabbit monoclonal, clone D7A5, from Cell Signaling Technology. 3777), anti human phospho Met (1:100 dilution, rabbit monoclonal, clone EP2367Y, from Abcam. ab68141). Fifty microliter of secondary antibody (Signal Stain Boost IHC Detection Reagent, HRP, Rabbit, Cell Signaling Technology. 81145) was added to the sections and incubated for 45 min at RT and washed 4 times in 1x PBS for 3 min each. Chromogenic development

was done by exposure of tissues to DAB substrate (DAB Peroxidase Substrate Kit; Vector Laboratories, SK-4100). Color development was monitored under light microscope. Appropriate negative antibody control (Isotype matched IgG) and/or tissue/cell line positive/negative controls were used accordingly. The tissues were counterstained using Papanicolaous solution (Merck. 60925301251730) and sections were mounted using DPX mountant medium. Each IHC result was evaluated by two independent experts and whenever there were differences in observation both experts came to a consensus Changes in the frequency of proliferating or dividing population of tumor cells in the explant tissues were evaluated using Ki-67 scoring (mean of total number of positive cells per field per section in triplicates)[2] . Phospho IHC score for EGFR and Met was analyzed based on combined value of percent positive area (0-100 scale) and intensity (0-3 scale)[3].

TUNEL assay

Apoptotic cells were detected by TUNEL method (ApopTag Peroxidase In Situ Apoptosis Detection Kit; Millipore. S7100). The effects of drugs in explants were determined based on viability of tissues at T0 time points (base line) and 72 h along with DMSO treated control. Briefly, the serial tissue sections were deparaffinized and rehydrated in graded ethanol. Antigen retrieval was done by incubating the tissue sections with Proteinase K (1:1000 dilution, Qiagen. 19131). Endogenous peroxidase was quenched by incubating sections with 3% H_2O_2. The sections were incubated with equilibration buffer for about 10 sec after which Terminal deoxynucleotidyltransferase (TdT) enzyme was added and incubated for 1 h at 37 °C in a humidified chamber. The reaction was terminated by washing the sections in stop/wash buffer for 10 min. Anti-digoxigenin conjugate was added to tissues and incubated for 30 min at RT. Chromogenic development was done by exposure of tissues to DAB substrate. The sections were counterstained using 0.5% methyl green.

Gene expression and microarray analysis

RNA later stabilized tissue samples were lysed using sample lysis buffer and total RNA was isolated. Total RNA was subsequently assessed for integrity by bio-analyzer and quantified by nanodrop for microarray experiments. RNA with 7 or above RIN value was used for microarray experiments. Approximately 50 ng of total RNA extracted from tumor samples was reverse transcribed to generate cy3 labeled amplified cRNA and was profiled using Agilent Kit and Agilent Sure Print G3 Human GE 8x60K Platform (Agilent Technologies. G4851B. http://www.chem.agilent.com). The array data obtained were normalized using feature extraction software and analyzed using GeneSpring Software. Data were expressed as fold differences (both for up-regulated and down-regulated genes) compared to the corresponding control. Any difference that was below 1.5 fold was considered as

insignificant for further validation. A heat map was generated and relationship (similarity of genes) was elucidated among different primary and xenograft tumor samples. Similarly, AS and TMP treated explant tissues were also subjected to gene expression profiling. Unsupervised array was used for generating a tree showing the relatedness of primary tumors with corresponding xenografts. From explants microarray both global expression pattern and specific signatures (i.e. TAM, Angiogenesis) were analyzed based on published[4] and commercially available data (SAB Bioscience, www.sabbiosciences.com). Statistical analysis was performed to distinguish the differentially expressed genes (at $P<0.05$) between different groups. Further association of P0 sample with corresponding HTX and association of gene expression patterns among AS+TMP and matched control groups were carried out using 3D principal component analysis (3D-PCA) (Agilent Technologies: http://www.chem.agilent.com). The raw and normalized microarray data are available at http://www.ncbi.nlm.nih.gov/geo/info/linking.html.

Mutational analysis

Genomic DNA was extracted from tumor tissues using QIAamp DNA Micro Kit (Qiagen. 56304) and subjected to PCR using region-specific primers to detect the mutational status of *KRAS* (codon numbers 12, 13, 61 and 146) by sequencing. Briefly, DNA fragment containing *KRAS* mutation hotspots were amplified with intron-based primers in reaction medium containing 2.5 mM MgCl2, 0.2 mM dNTPs, 1 µM of each primer, and 0.5 U of Phusion Taq (Life Technologies. F-530S) in a total volume of 50 µl. The PCR was carried out at 95 °C for 5 min, followed by 25 cycles at 95 °C for 30 sec; 60 °C for 30 sec; 72 °C for 30 sec with a final extension for 5 min. The PCR products were resolved on 1.5% agarose gels. The amplicons were excised and purified using QIAquick gel extraction kit (Qiagen. 28704) according to manufacturer's protocol and processed for Sanger sequencing.

Quantitative PCR

RNA later stabilized tissues were lysed using micro-dismembrator (Sartorius) and total RNA was isolated from pulverized tissues using an RNeasy Micro kit (Qiagen. 74004). The quality of RNA was assessed by nanodrop. A total 250 ng of RNA was reverse-transcribed using the High-Capacity cDNA Reverse Transcription kit (Applied Biosytems. 4368813) according to the manufacturer's protocol. Each PCR was carried out in 20 µl of a reaction mix, containing 5 µl of SYBR select master mix (Applied Biosystems; 4472908), 200 nM primers and 100 ng of cDNA. The following PCR conditions used in this experiment: UDG activation at 50 °C for 2 min followed by Amplitaq Fast DNA polymerase activation at 95 °C for 2 min, denaturation at 95 °C for 15 sec, annealing at appropriate temperature for 15 sec and

extension at 72 °C for 1 min. The amplified products were run on 1% agarose gel to verify the product size.

Exome sequence analysis

Genomic DNAs were isolated from primary HNSCC tumors using DNAeasy Tissue Kit according to manufacturer's protocol (Qiagen. 56304). The quality of DNA was performed by spectrophotometry and DNA with A260/A280 ratio between 1.7 and 1.9 was used for further processing. Additionally, the DNA integrity was also checked by *EcoRI* digestion followed by gel analysis. Following quality check, exome sequencing of the DNAs was conducted at BGI Americas (Philadelphia, PA). The qualified genomic sample was randomly fragmented with a base pair peak of 150-200 bp followed by adaptor ligation on both ends of the fragment. The adaptor ligated templates were purified by Agencourt AMPure SPRI beads. Fragments with insert size ~200 bp were excised. Extracted DNA was amplified by ligation mediated PCR, purified and hybridized to the SureSelect Biotinylated RNA library for enrichment. Hybridized fragments were bound to the streptavidin beads whereas non-hybridized fragments were washed out after 24 h. Captured LM-PCR products were subjected to Agilent 2100 Bioanalyzer to estimate the magnitude of enrichment. Each captured library was then loaded on Hiseq2000 platform, and high-throughput sequencing was performed for each captured library independently to ensure that each sample meets the desired average fold-coverage. Raw image files were processed by Illumina base calling Software 1.7 for base calling with default parameters and the sequences of each individual were generated as 90 bp paired-end reads. Differences in the broad mutations' spectrum in clinical responder and non-responder groups and corresponding HTXs were determined. Circos plots were generated to show the patterns of these mutations in pre and post grafts. Sequence data was aligned to the hg19 (b37) reference genome with the Burrows-Wheeler Aligner[5] with parameters -q 5 -l 32 -k 2 -t 4 -o 1. Aligned data was sorted, normalized, mate-fixed, duplicate-marked, and indexed with SAM tools and Picard tools. Base quality score recalibration was achieved with the Genome Analysis Toolkit[6]. Somatic mutations were called with MuTect then filtered and annotated to genes with Oncotator [7]. Significantly mutated genes/gene sets linked to specific pathways were determined for both primary and HTXs and aligned in a pair wise fashion. The tumor driver pathway analysis was performed to dissect the candidate drivers for these tumors.

Supplementary References:

1. Shackelford, D.B., *et al.* LKB1 inactivation dictates therapeutic response of non-small cell lung cancer to the metabolism drug phenformin. *Cancer Cell* **23**, 143-158 (2013).
2. Vaira, V., *et al.* Preclinical model of organotypic culture for pharmacodynamic profiling of human tumors. *Proc Natl Acad Sci U S A* **107**, 8352-8356 (2010).

3. Benedettini, E., *et al.* Met activation in non-small cell lung cancer is associated with de novo resistance to EGFR inhibitors and the development of brain metastasis. *Am J Pathol* **177**, 415-423 (2010).
4. Straussman, R., *et al.* Tumour micro-environment elicits innate resistance to RAF inhibitors through HGF secretion. *Nature* **487**, 500-504 (2012).
5. Li, H. & Durbin, R. Fast and accurate short read alignment with Burrows-Wheeler transform. *Bioinformatics* **25**, 1754-1760 (2009).
6. McKenna, A., *et al.* The Genome Analysis Toolkit: a MapReduce framework for analyzing next-generation DNA sequencing data. *Genome Res* **20**, 1297-1303 (2010).
7. DePristo, M.A., *et al.* A framework for variation discovery and genotyping using next-generation DNA sequencing data. *Nat Genet* **43**, 491-498 (2011).
